# Supplementary material for: Frailty Levels In Geriatric Hospital paTients (FLIGHT)—the prevalence of frailty among geriatric populations within hospital ward settings: a systematic review protocol
Source: BMJ Open. 2019 Aug 24;9(8):e030147. doi: 10.1136/bmjopen-2019-030147 (PMC6720252; doi:10.1136/bmjopen-2019-030147)
Supplement: Supplementary data [file bmjopen-2019-030147supp001.pdf]

Search Strategy:

Ovid Search Strategy

1. Frail$.ti.ab.
2. Prevalence.ti,ab.
3. Percent$.ti,ab.
4. "were frail".ti,ab.
5. "considered frail".ti,ab.
6. Hospital$.ti,ab.
7. Ward.ti,ab.
8. Department.ti,ab.
9. Surg*.ti,ab.
10. Unit.ti,ab.
11. Geriatr*.tx.
12. "older adult*".tx.
13. Elder$.tx.
14. Retire*.tx.
15. Old$.tx.
16. Patient$.tx.
17. "community-dwelling".ti,ab.
18. 2 OR 3 OR 4 OR 5
19. 6 OR 7 OR 8 OR 9 OR 10
20. 11 OR 12 OR 13 OR 14 OR 15 OR 16
21. 1 AND 18 AND 19 AND 20
22. 21 NOT 17

Scopus Search Strategy

((((TITLE-ABS-KEY(frail*)) AND (TITLE-ABS-KEY(Prevalence)) OR (TITLE-ABS-KEY(Percent*)) OR (TITLE-ABS-KEY ("were frail")) OR (TITLE-ABS-KEY ("considered frail"))) AND (((TITLE-ABS-KEY(Hospital*)) OR (TITLE-ABS-KEY(Ward)) OR (TITLE-ABS-KEY(Department)) OR (TITLE-ABS-KEY(surg*)) OR (TITLE-ABS-KEY(unit))))) AND ((ALL(Geriatr*)) OR (ALL("older adult*")) OR (ALL(Elder*)) OR (ALL(retire*)) OR (ALL(old)) OR (ALL(older)) OR (ALL(Patient*)))) AND NOT (TITLE-ABS-KEY("community-dwelling"))

Web of Science Search Strategy

1. TS = Frail*
2. TS = Prevalence
3. TS = Percent*
4. TS = "were frail"
5. TS = "considered frail"
6. TS = Hospital*
7. TS = Ward
8. TS = Department
9. TS = Surg*
10. TS = Unit
11. TS = Geriatr*
12. TS = "older adult"
13. TS = Elder*
14. TS = Retir*
15. TS = Old*
16. TS = Patient*
17. TS = "community-dwelling"
18. #2 OR #3 OR #4 OR #5

19. #6 OR #7 OR #8 OR #9 OR #10
20. #11 OR #12 OR #13 OR #14 OR #15 OR #16
21. #1 AND #18 AND #19 AND #20
22. #21 NOT #17

CINAHL PLUS Search Strategy

1. AB frail*
2. AB prevalence OR AN Percent* OR AB "were frail" OR AB "considered frail"
3. AB Hospital* OR AB Ward OR AB Department OR AB Surg* OR AB Unit
4. AB Geriatr* OR AB "older adult" OR AB Elder* OR AB Retir* OR AB OLD* OR AB Patient*
5. S1 AND S2 AND S3 AND S4

Cochrane Library Search Strategy

1. frail*:ti,ab,kw (Word variations have been searched)
2. prevalence:ti,ab,kw or percent*:ti,ab,kw or "were frail":ti,ab,kw or "considered frail":ti.ab.kw (Word variations have been searched)
3. hospital*:ti,ab,kw or ward:ti,ab,kw or department:ti,ab,kw or surg*:ti,ab,kw or unit:ti,ab,kw (Word variations have been searched)
4. Geriatr*:ti,ab,kw or "older adult":ti,ab,kw or Elder*:ti,ab,kw or Retir*:ti,ab,kw or Old*:ti,ab,kw (Word variations have been searched)
5. Patient*:ti,ab,kw (Word variations have been searched)
6. #4 OR #5
7. #1 AND #2 AND #3 AND #6
