# Supplementary material for: Frailty Levels In Geriatric Hospital paTients (FLIGHT)—the prevalence of frailty among geriatric populations within hospital ward settings: a systematic review protocol
Source: BMJ Open. 2019 Aug 24;9(8):e030147. doi: 10.1136/bmjopen-2019-030147 (PMC6720252; doi:10.1136/bmjopen-2019-030147)
Supplement: Supplementary data [file bmjopen-2019-030147supp002.pdf]

Frailty Levels In Geriatric Hospital in-paTients (FLIGHT)
Systematic Review Search Strategy Screening form
PANINI (Physical Activity and Nutritional INfluences in Ageing) project
University of Birmingham
2016 - 2019

UNIVERSITY OF BIRMINGHAM

"The prevalence of frailty amongst geriatric populations within hospital ward settings: A systematic review"

Inclusion criteria - All studies must:

- have a minimum age of ≥ 65 years
- use a clearly defined and validated operational definition for the classification of frailty
- either assess (or attempt to assess) the whole ward, department, unit, hospital, or clinical population, or employ some form of randomised selection of participants
- occur within a hospital setting, in, or including hospital in-patients*
- report the prevalence of frailty or provide sufficient data to allow the calculation of the prevalence of frailty.

* If a study examines a mixed cohort, only data relating to hospital in-patients will be included.

Exclusion criteria:

- studies not written in English
- studies where the sample are non-hospital in-patients (i.e. outpatients, day patients or community-dwelling individuals)

Systematic Review - Screening procedure

1). Import attached RIS file into your reference manager software (preferably EndNote X8.2)

2). Once imported, scan all title and abstracts for eligibility against the inclusion / exclusion criteria above.

3). Move all studies identified as potentially eligible based on title and abstract into a separate group (EndNote), Or folder (RefWorks, Mendeley).

4). Screen full text of identified studies to determine eligibility.

5). Move all eligible studies into separate group / folder.

6). Make note of excluded studies and reasons for their exclusion based on eligibility criteria, in the attached excel file.

5). Compare identified studies.

6). If all reviewers identify the exact same studies, with no discrepancies, this is the end of the initial screening process for the systematic review.

7). If there are differences in the studies identified by different reviewers - discuss until resolution is determined. In the event a unanimous consensus cannot be met by the three reviewers, the majority consensus will be taken, and a note made of this.

Doody P, *et al. BMJ Open* 2019; 9:e030147. doi: 10.1136/bmjopen-2019-030147
