# Supplementary material for: Frailty Levels In Geriatric Hospital paTients (FLIGHT)—the prevalence of frailty among geriatric populations within hospital ward settings: a systematic review protocol
Source: BMJ Open. 2019 Aug 24;9(8):e030147. doi: 10.1136/bmjopen-2019-030147 (PMC6720252; doi:10.1136/bmjopen-2019-030147)
Supplement: Supplementary data [file bmjopen-2019-030147supp003.pdf]

Author	Title	Initial studies included from full text screening (prior to reviewer discussion)		
		Reviewer 1 (PD)	Reviewer 2 (JA)	Reviewer 3 (EA)

Conclusion of discussion	Decision	Response received from author	Outcome

Key

**Unanimous consensus inclusion**	
**Majority consensus inclusion**	
**Minority consensus inclusion / contact author***	*In event where either there is not majority consensus inclusion or exclusion i.e. one reviewer wishes to include and at least one other wishes to seek further information, or two reviewers wish to seek futher information
**Majority consensus exclusion****	** = Reasons for all excluded studies are given in exclusion form
**Unanimous consensus exclusion****	** = Reasons for all excluded studies are given in exclusion form
Unanimous consensus contact author	
? = Contact author***	** = Reasons for all instances of contacting the study author for clarification or futher information to assess eligibility are outlined in contact author form

✓ = Included

x = Excluded
