# Supplementary material for: Frailty Levels In Geriatric Hospital paTients (FLIGHT)—the prevalence of frailty among geriatric populations within hospital ward settings: a systematic review protocol
Source: BMJ Open. 2019 Aug 24;9(8):e030147. doi: 10.1136/bmjopen-2019-030147 (PMC6720252; doi:10.1136/bmjopen-2019-030147)
Supplement: Supplementary data [file bmjopen-2019-030147supp004.pdf]

	Study details						Study Methods													
										Subject characteristics										
Study	Author	Year of Publication	Study title	Journal of publication	Aim	Setting	Ward / Department / Unit / Hospital / Clinical population type	Study design	Recruitment duration	Age of participants (mean +/-SD)	Age of participants (range)	Sex (proportion male / female participants)	Country / location	Continent	5 year average GDP per capita PPP (current international $) (years proceeding the study) (International Monetary Fund data)	5 year average healthcare expenditure per capita PPP (current international $) (years proceeding the study) (World Health Organisation data)	Sample size (n)	Diagnosis / Prevalent morbidity (if applicable)	Any other relevant characteristic	Criteria utilised for the operational definition of frailty
Study 2																				
Study 3																				
Study 4																				
Study 5																				
Study 6																				
Study 7																				
Study 8																				
Study 9																				
Study 10																				
Study 11																				
Study 12																				
Study 13																				
Study 14																				
Study 15																				
Study 16																				
Study 17																				
Study 18																				
Study 19																				
Study 20																				
Study 21																				
Study 22																				
Study 23																				

Planned stratified analysis

Results																					
Number of frail participants	Number of "pre-frail" participants	Number of robust / non-frail	Prevalence of frailty	Prevalence of "pre-frailty"	Prevalence of robust/non frail	Number of Male participants	Number of frail Male participants	Number of pre-frail Male participants	Number of non-frail / robust Male participants	Prevalence of frailty in Male participants	Prevalence of pre-frailty in Male participants	Prevalence of robust / non-frailty Male participants	Number of Female participants	Number of frail Female partcipants	Number of pre-frail Female participants	Number of non-frail / robust Female participants	Prevalence of frailty in Female participants	Prevalence of pre-frailty in Female participants	Prevalence of robust / non-frialty Female participants	Relevant authors comments	Relevant reviewers comments

					5 year average GDP per capita PPP (current international $) (years proceeding the study*) (International Monetary Fund data)																		
Author	Year of Publication	Country / location	Recruitment start date	Recruitment end date	Year 5	Year 4	Year 3	Year 2	Year 1	Additional Year 1	Additional year 2	Additional year 3	Additional year 4	Additional year 5	Additional year 6	Additional year 7	Additional year 8	Additional year 9	Additional year 10	Additional year 11	Additional year 12	Years	5 year average GDP per capita PPP (current international $) (years proceeding the study*) (Internation Monetary Fund data)

*5 years prior to commencement of data collection for the study. Each calender year of the study will also be included provided recruitment continues through to > 6 months in the proceeding year.

					5 year average healthcare expenditure per capita PPP (current international $) (years proceeding the study) (World Health Organisation data)																			
Author	Year of Publication	Country / location	Recruitment start date	Recruitment end date	Year 5	Year 4	Year 3	Year 2	Year 1	Additional Year 1	Additional year 2	Additional year 3	Additional year 4	Additional year 5	Additional year 6	Additional year 7	Additional year 8	Additional year 9	Additional year 10	Additional year 11	Additional year 12	Years	5 year average health care expenditure per capita PPP (current international $) (years proceeding the study*) (World Health Organisation data)	

*5 years prior to commencement of data collection for the study. Each calender year of the study will also be included provided recruitment continues through to > 6 months in the proceeding year.
