# Supplementary material for: Frailty Levels In Geriatric Hospital paTients (FLIGHT)—the prevalence of frailty among geriatric populations within hospital ward settings: a systematic review protocol
Source: BMJ Open. 2019 Aug 24;9(8):e030147. doi: 10.1136/bmjopen-2019-030147 (PMC6720252; doi:10.1136/bmjopen-2019-030147)
Supplement: Author's manuscript [file bmjopen-2019-030147.draft_revisions.pdf]

BMJ Open

BMJ Open is committed to open peer review. As part of this commitment we make the peer review history of every article we publish publicly available.

When an article is published we post the peer reviewers' comments and the authors' responses online. We also post the versions of the paper that were used during peer review. These are the versions that the peer review comments apply to.

The versions of the paper that follow are the versions that were submitted during the peer review process. They are not the versions of record or the final published versions. They should not be cited or distributed as the published version of this manuscript.

BMJ Open is an open access journal and the full, final, typeset and author-corrected version of record of the manuscript is available on our site with no access controls, subscription charges or pay-per-view fees (http://bmjopen.bmj.com).

If you have any questions on BMJ Open's open peer review process please email
info.bmjopen@bmj.com

BMJ Open

Frailty Levels in Geriatric Hospital paTients (FLIGHT) - The prevalence of frailty amongst geriatric populations within hospital ward settings: A systematic review protocol

Journal:	*BMJ Open*
Manuscript ID	bmjopen-2019-030147
Article Type:	Protocol
Date Submitted by the Author:	28-Feb-2019

Complete List of Authors:	Doody, Paul; University of Birmingham, School of Sport, Exercise and Rehabilitation Sciences<br>Aunger, Justin; University of Birmingham, School of Sport, Exercise and Rehabilitation Sciences<br>Asamane, Evans; University of Birmingham, School of Sport, Exercise and Rehabilitation Sciences<br>Greig, Carolyn; School of Sport, Exercise and Rehabilitation Sciences, University of Birmingham, Edgbaston, Birmingham, B15 2TT, UK; MRC-Arthritis Research UK Centre for Musculoskeletal Ageing Research<br>Lord, Janet; University of Birmingham, NIHR Surgical Reconstruction & Microbiology Research Centre<br>Whittaker, Anna; University of Birmingham, School of Sport, Exercise and Rehabilitation Sciences; University of Stirling

Keywords:	Frail, Geriatric, inpatient, older adult, prevalence, systematic review

SCHOLARONE™
Manuscripts

Protocol_Manuscript_V1.0_07/02/2019

Frailty Levels in Geriatric Hospital paTients (FLIGHT) - The prevalence of frailty amongst geriatric populations within hospital ward settings: A systematic review protocol

Paul Doody[1], Justin A. Aunger[1], Evans A. Asamane[1], Carolyn A. Greig[1,2,3], Janet M. Lord[2], Anna C. Whittaker[1,4]

[1] School of Sport, Exercise and Rehabilitation Sciences, University of Birmingham, United Kingdom

[2] Institute of Inflammation and Ageing MRC-ARUK Centre for Musculo-skeletal Ageing Research, University of Birmingham, United Kingdom

[3] NIHR Birmingham Biomedical Research Centre, University Hospitals Birmingham NHS Foundation Trust and University of Birmingham, United Kingdom

[4] Faculty of Health Sciences and Sport, University of Stirling, United Kingdom

Corresponding author: Paul Doody (p.d.doody@bham.ac.uk, +44 (0) 121 414 4125), School of Sport Exercise and Rehabilitation Sciences (G35), University of Birmingham, United Kingdom

For peer review only

Abstract

Introduction: Frailty is a common and clinically significant condition in geriatric populations, associated with adverse health outcomes such as hospitalisation, disability, and mortality. Although there are systematic reviews/meta-analyses assessing the prevalence of frailty in community-dwelling older adults, nursing home residents, and cancer and general surgery patients, there are none assessing the overall prevalence of frailty in geriatric hospital inpatients.

Methods and analysis: This review will systematically search and analyse the prevalence of frailty within geriatric hospital inpatients within the literature. A search will be employed on the platforms of Ovid, Web of Science, and databases of CINAHL Plus, SCOPUS, and the Cochrane Library. Any observational or experimental study design which utilises a validated operational definition of frailty, reports the prevalence of frailty, has a minimum age ≥ 65 years, attempts to assess the whole ward/clinical population, and occurs in hospital inpatients, will be included. Title and abstract and full-text screenings will be conducted by three reviewers. Methodological quality of eligible studies will be assessed utilising the Joanna Briggs Institute critical appraisal tool. Data extraction will be performed by two reviewers. If sufficient data are available, a meta-analysis synthesising pooled estimates of the prevalence of frailty and pre-frailty, as well as the prevalence of frailty stratified by age, sex, frailty definition, prevalent morbidities, ward type, and location, among older hospitalised in-patients will be conducted. Clinical heterogeneity will be assessed by two reviewers. Statistical heterogeneity will be assessed through a Cochran Q test, and an I^2 test performed to assess its magnitude.

Ethics and dissemination: Ethical approval was not required as primary data will not be collected. Findings will be disseminated through publication in peer reviewed open access scientific journals, public engagement events, conference presentations, and social media**.**

Trial Registration number: This study has been registered on PROSPERO (registration number 79202).

Strengths and limitations of this study:

For peer review only - http://bmjopen.bmj.com/site/about/guidelines.xhtml

For peer review only

- First review to systematically assess the overall prevalence of frailty in geriatric hospital inpatients
- Will seek to provide stratified analysis of the prevalence of frailty based on age, sex, frailty definition, prevalent morbidities, ward type and location
- Three independent reviewers; ensuring high internal reliability and consistency
- Will include only studies for which the full text is available in English
- Will exclusively assesses the prevalence of frailty in geriatric hospital inpatients
- **Keywords**: department; frail; geriatric; hospital; inpatient; meta-analysis; older adult; prevalence; systematic review; ward.

For peer review only

Introduction

Frailty is a common and clinically significant condition within geriatric populations (Rodriguez-Mañas, Fried, 2015), predominantly due to its association with adverse health outcomes such as hospitalization, disability and mortality (Fried et al., 2001, Gill et al., 2006, Sternberg et al., 2011, Clegg et al., 2013, Rodriguez-Mañas, Fried, 2015, Sourial et al., 2013). Although there are systematic reviews and meta-analysis assessing the prevalence of frailty amongst community-dwelling older adults (Collard et al., 2012, Siriwardhana et al., 2018, Kojima et al., 2017, Verlaan et al., 2017), nursing home residents (Kojima, 2015), and cancer (Handforth et al., 2015) and general surgery patients (Hewitt et al., 2018), presently there are no systematic reviews or meta-analysis which assess the overall prevalence of frailty among geriatric hospital inpatients. This constitutes an important gap in the literature which needs to be addressed and has important consequences. Such consequences include the tailoring of services within this setting to the needs of service users, for example, the potential implementation of exercise rehabilitation treatments within this setting for this cohort; with physical activity and exercise being proposed as potentially offering the best form of treatment for frail older adults (Theou et al., 2011), and shown to be capable of reducing, and even reversing frailty within older adults (Fiatarone et al., 1994, Tarazona-Santabalbina et al., 2016). Through providing a highly detailed analysis of the prevalence of frailty amongst older population within this setting, this review has the potential to aid in the facilitation of improvements in the planning and orientation of organisational structures and resources, to meet the needs of this population, and enhance the care of frail older adults in inpatient hospital settings.

Methods and Design

Review Aim:

The aim of this review is to systematically search and analyse the prevalence of frailty amongst geriatric populations within inpatient hospital settings within the literature. If a meta-analysis proves possible, the aim of this study is also to synthesise pooled estimates of the prevalence of frailty and pre-frailty, as well as the

For peer review only - http://bmjopen.bmj.com/site/about/guidelines.xhtml

prevalence of frailty stratified by age, sex, frailty definition, prevalent morbidities, ward type and location, among hospital in-patients.

Review Objectives:

1) To identify and compare studies reporting the prevalence of frailty within hospital ward settings.
2) To combine the extracted data to calculate the pooled overall prevalence of frailty in hospitalised geriatric in-patients.
3) To perform stratified analysis of the prevalence of frailty based on age, sex, frailty definition, prevalent morbidity and ward type in order to assess the relationship between frailty and these factors.

Eligibility criteria:

Inclusion criteria: all studies must have a minimum age of ≥ 65 years, use a clearly defined and validated operational definition for the classification of frailty, either assess (or attempt to assess) the whole ward, department, unit, hospital or specific clinical population, or employ some form of randomised selection of participants, occur within a hospital setting, in, or including, hospital in-patients, report the prevalence of frailty or provide sufficient data to allow the calculation of the prevalence of frailty. If a study examines a mixed cohort, only data relating to hospital in-patients will be included in the review.

Exclusion criteria: all studies not written in English, studies where the sample are non-hospital in-patients (i.e. outpatients, day patients or community-dwelling individuals).

Information sources:

Searches will be conducted on the platforms of Ovid (incorporating the databases of Journals @Ovid full text, EMBASE, CAB abstracts, Ovid MEDLINE ® In process and other non-indexed citations, Ovid MEDLINE ®, and PyschINFO) and Web of Science (incorporating the databases of Science Citation Index

For peer review only - http://bmjopen.bmj.com/site/about/guidelines.xhtml

Expanded (SCI-Expanded), Conference Proceedings Citation Index – Science (CRI-S), and Emerging Sources Citation Index (ESCI)), and the databases of CINAHL Plus, SCOPUS, and the Cochrane Library databases (the Cochrane Database of Systematic Reviews (CDSR), the Cochrane Central Register of Controlled Trials (CENTRAL), the Cochrane Methodology Register (CMR), the Database of Abstracts of Reviews of Effect (DARE), Health Technology Assessment database (HTA) and the NHS Economic Evaluation Database (EED)).

Types of studies:

Any form of observational or experimental study design which assesses the prevalence of frailty and meets the above eligibility criteria. For longitudinal observational studies, and experimental studies, frailty scores and additional data will be extracted from baseline data, provided baseline data meets the above eligibility criteria.

Search Strategy:

The search strategy will be conducted on the two platforms of Ovid and Web of Science, as well as the databases of SCOPUS, CINAHL Plus, and the Cochrane Library databases (Appendix 1).

Screening:

Prior to the commencement of title and abstract screening by the three independent reviewers, duplicates will be removed utilising EndNote X8.2. The reduced list of studies will be manually screened for the removal of any remaining duplicates. All reviewers will be provided with an instructional screening form (Appendix 2), and a .ris file containing all studies captured within database searches. The screening form will list the eligibility criteria and instructions on setting up the .ris file for screening within a reference manager.

For peer review only - http://bmjopen.bmj.com/site/about/guidelines.xhtml

For peer review only

The title and abstract of all studies will then be independently screened by the three reviewers, with each reviewer placing potentially eligible studies into a separate folder. Upon completion, potentially eligible studies from all three reviewers will be placed into a "master folder" and the results collated. Duplicates will be removed, leaving the final combined list of studies for the full text screening phase. All reviewers will then independently screen the full text of remaining studies utilising the screening form and maintain separate files for included and excluded studies (including reasons), as well as for studies for which the reviewer feels the need to contact the authors for clarification or additional information.

Upon completion, a full text screening master file (Appendix 3) will be formulated by the lead reviewer displaying each reviewer's full text screening decision for each study. All three reviewers will then meet to discuss the decisions of each study and endeavour to come to an agreement on studies for which there is not initial unanimous consensus. During this process a full list of included and excluded studies (with reasons), and studies for which reviewers agree to contact authors for additional information or clarification will be formed by the lead reviewer. The lead reviewer will then contact study authors and, upon receipt of clarification or additional information, will meet with reviewers to discuss the inclusion/exclusion of the study.

Assessment of methodological quality:

The quality of eligible studies from full text screening will be assessed by two reviewers independently using the Joanna Briggs Institute (JBI) critical appraisal tool for studies reporting prevalence data (Munn et al., 2014) (Appendix 4). In the event of any discrepancies between the two reviewers, a consensus will be attempted to be reached by discussion. In the event a full consensus cannot be reached between the two reviewers after an exhaustive discussion, the opinion of a third reviewer will be obtained, and the proceeding majority consensus will be taken.

Data extraction:

For peer review only - http://bmjopen.bmj.com/site/about/guidelines.xhtml

Data extraction will be performed by two reviewers independently. In the event of any discrepancies between the two reviewers, a consensus will be attempted to be reached by discussion. In the event that a full consensus cannot be reached between the two reviewers after an exhaustive discussion, the opinion of a third reviewer will be obtained, and the proceeding majority consensus will be taken.

The following data, where available, will be extracted from all eligible studies (see Appendix 5 for template). If any data are not immediately available, the authors of the studies in question will be contacted in an attempt to retrieve all applicable data:

Study details: authors, year of publication, study title, journal of publication, aim. Study methods: setting, ward/department/unit/hospital type/clinical population, study design, study duration, subject characteristics (age of participants (mean and standard deviation, range)), sex (proportion of male / female participants), country / location, sample size, diagnosis / prevalent morbidity (if applicable), and any other relevant characteristics). Criteria utilised for the operational definition of frailty, Dependent variables, and Methods of data analysis. Results: prevalence of frailty, prevalence of pre-frailty, prevalence of robust/non-frailty, and finally authors comments and reviewers' comments.

Data synthesis:

Quantitative synthesis (Meta-Analysis): If a sufficient quantity of identified studies are comparable, a meta-analysis, pooling the aggregated data from each study, will be performed. Clinical heterogeneity will be assessed by two reviewers based on their judgement of the available data and any disagreements will be discussed thoroughly with the aim of reaching a unanimous consensus. If a unanimous consensus cannot be reached, the opinion of a third reviewer will be sought, and the proceeding majority consensus will be taken. Statistical heterogeneity will be assessed through the utilisation of a Cochran Q test and considered present at $p < .05$. An I^2 test will be performed in order to assess the magnitude of this heterogeneity, with I^2 values of 25%, 50% and 75% being considered low, moderate and high respectively. If the Cochrane Q statistic test detected statistically significant heterogeneity, combined with the researcher's assessment, a randomised-

For peer review only - http://bmjopen.bmj.com/site/about/guidelines.xhtml

effects model will be utilised. Given the nature of this review and in particular its overall aim, combined with the eligible studies identified in preliminary searches, it is likely the initial quantitative synthesis will utilise a random-effects model.

Stratified analysis will also be conducted according to age (65 – 74 years versus 75+ years to assess younger versus older old), sex, frailty definition, ward type, prevalent morbidity and location where possible. Similarly, a random-effects model will be utilised to synthesise pooled estimates of the prevalence of frailty stratified by these criteria (although there is more of a likelihood that a fixed effects model could potentially be utilised within these analyses, in comparison to the initial analysis, given the nature of stratified analysis).

Qualitative synthesis: if a meta-analysis is not possible based on the nature of the studies and the data available, a more thorough systematic narrative analysis will be conducted, with findings presented in both textual and tabular formats.

Ethics and Dissemination

Formal ethical approval was not required for this review as primary data will not be collected. The findings of this study will be disseminated through publication in the form of scientific papers in peer reviewed open access scientific journals, public engagement events within the United Kingdom and Europe, online via social media (Twitter, Instagram) and the PANINI project website (Whittaker et al., 2018, University of Birmingham,), and presentation at conferences within the UK and internationally.

Funding

This review has been funded by the European Commission's Horizon 2020 research and innovation programme under the Marie Sklodowska-Curie grant agreement (675003); of which PD, EA and JA are

For peer review only

Marie Sklodowska-Curie Doctoral Research Fellows, AW, JL and CG doctoral supervisors, and AW the grants Principal Investigator.

Author contributions

PD is guarantor and lead reviewer. PD designed the systematic review protocol, conducted the literature searches and prepared this manuscript for publication, with supervision, input and feedback from AW, CG and JL. EA and JA are independent reviewers for title and abstract and full text screenings. JA will also act as independent data extractor for included studies. All authors have read and approved the final manuscript.

Conflicts of interest:

The authors of this review report no known conflicts of interest.

Acknowledgements:

The authors of this review would like to thank and acknowledge Ms. Lynne Harris (Subject advisor of the Main Library at the University of Birmingham, United Kingdom) for her assistance during the formulation of the search strategy utilised within this systematic review.

Word count (excluding references and cover page): 2282

For peer review only - http://bmjopen.bmj.com/site/about/guidelines.xhtml

References:

Clegg, A., Young, J., Iliffe, S., Rikkert, M.O. & Rockwood, K. 2013, "Frailty in elderly people", *The Lancet,* vol. 381, no. 9868, pp. 752-762.

Collard, R.M., Boter, H., Schoevers, R.A. & Oude Voshaar, R.C. 2012, "Prevalence of frailty in community-dwelling older persons: a systematic review", *Journal of the American Geriatrics Society,* vol. 60, no. 8, pp. 1487-1492.

Fiatarone, M.A., O'Neill, E.F., Ryan, N.D., Clements, K.M., Solares, G.R., Nelson, M.E., Roberts, S.B., Kehayias, J.J., Lipsitz, L.A. & Evans, W.J. 1994, "Exercise training and nutritional supplementation for physical frailty in very elderly people", *New England Journal of Medicine,* vol. 330, no. 25, pp. 1769-1775.

Fried, L.P., Tangen, C.M., Walston, J., Newman, A.B., Hirsch, C., Gottdiener, J., Seeman, T., Tracy, R., Kop, W.J. & Burke, G. 2001, "Frailty in older adults evidence for a phenotype", *The Journals of Gerontology Series A: Biological Sciences and Medical Sciences,* vol. 56, no. 3, pp. M157.

Gill, T.M., Gahbauer, E.A., Allore, H.G. & Han, L. 2006, "Transitions between frailty states among community-living older persons", *Archives of Internal Medicine,* vol. 166, no. 4, pp. 418-423.

Handforth, C., Clegg, A., Young, C., Simpkins, S., Seymour, M.T., Selby, P.J. & Young, J. 2015, "The prevalence and outcomes of frailty in older cancer patients: a systematic review", *Annals of Oncology,* vol. 26, no. 6, pp. 1091-1101.

Hewitt, J., Long, S., Carter, B., Bach, S., McCarthy, K. & Clegg, A. 2018, "The prevalence of frailty and its association with clinical outcomes in general surgery: a systematic review and meta-analysis", *Age and Ageing,* vol. 47, no. 6, pp. 793-800.

Kojima, G. 2015, "Prevalence of frailty in nursing homes: A systematic review and meta-analysis", *Journal of the American Medical Directors Association,* vol. 16, no. 11, pp. 940-945.

Kojima, G., Iliffe, S., Taniguchi, Y., Shimada, H., Rakugi, H. & Walters, K. 2017, "Prevalence of frailty in Japan: A systematic review and meta-analysis", *Journal of epidemiology,* vol. 27, no. 8, pp. 347-353.

Munn, Z., Moola, S., Riitano, D. & Lisy, K. 2014, "The development of a critical appraisal tool for use in systematic reviews addressing questions of prevalence", *International journal of health policy and management,* vol. 3, no. 3, pp. 123.

Rodriguez-Mañas, L. & Fried, L.P. 2015, "Frailty in the clinical scenario", *The Lancet,* vol. 385, no. 9968, pp. e9.

Siriwardhana, D.D., Hardoon, S., Rait, G., Weerasinghe, M.C. & Walters, K.R. 2018, "Prevalence of frailty and prefrailty among community-dwelling older adults in low-income and middle-income countries: a systematic review and meta-analysis", *BMJ open,* vol. 8, no. 3, pp. e018195.

Sourial, N., Bergman, H., Karunananthan, S., Wolfson, C., Payette, H., Gutierrez-Robledo, L.M., Béland, F., Fletcher, J.D. & Guralnik, J. 2013, "Implementing Frailty Into Clinical Practice: A Cautionary Tale", *The Journals of Gerontology Series A: Biological Sciences and Medical Sciences,* , pp. glt053.

Sternberg, S.A., Schwartz, A.W., Karunananthan, S., Bergman, H. & Mark Clarfield, A. 2011, "The identification of frailty: a systematic literature review", *Journal of the American Geriatrics Society,* vol. 59, no. 11, pp. 2129-2138.

Tarazona-Santabalbina, F.J., Gmez-Cabrera, M.C., Prez-Ros, P., Martnez-Arnau, F.M., Cabo, H., Tsaparas, K., Salvador-Pascual, A., Rodriguez-Maas, L. & Via, J. 2016, "A multicomponent exercise intervention that reverses

For peer review only - http://bmjopen.bmj.com/site/about/guidelines.xhtml

frailty and improves cognition, emotion, and social networking in the community-dwelling frail elderly: a randomized clinical trial", *Journal of the American Medical Directors Association,* vol. 17, no. 5, pp. 426-433.

Theou, O., Stathokostas, L., Roland, K.P., Jakobi, J.M., Patterson, C., Vandervoort, A.A. & Jones, G.R. 2011, "The effectiveness of exercise interventions for the management of frailty: a systematic review", *Journal of aging research,* vol. 2011.

University of Birmingham , *Physical Activity and Nutritional INfluences In ageing (PANINI) *. Available: http://www.birmingham.ac.uk/generic/panini/index.aspx [2019, 6th February].

Verlaan, S., Ligthart-Melis, G.C., Wijers, S.L., Cederholm, T., Maier, A.B. & de van der Schueren, Marian AE 2017, "High prevalence of physical frailty among community-dwelling malnourished older adults–a systematic review and meta-analysis", *Journal of the American Medical Directors Association,* vol. 18, no. 5, pp. 374-382.

Whittaker, A.C., Delledonne, M., Finni, T., Garagnani, P., Greig, C., Kallen, V., Kokko, K., Lord, J., Maier, A.B. & Meskers, C.G. 2018, "Physical Activity and Nutrition INfluences In ageing (PANINI): consortium mission statement", *Aging clinical and experimental research,* , pp. 1-8.

For peer review only

Search Strategy:

Ovid Search Strategy

1. Frail$.ti.ab.
2. Prevalence.ti,ab.
3. Percent$.ti,ab.
4. "were frail".ti,ab.
5. "considered frail".ti,ab.
6. Hospital$.ti,ab.
7. Ward.ti,ab.
8. Department.ti,ab.
9. Surg*.ti,ab.
10. Unit.ti,ab.
11. Geriatr*.tx.
12. "older adult*".tx.
13. Elder$.tx.
14. Retire*.tx.
15. Old$.tx.
16. Patient$.tx.
17. "community-dwelling".ti,ab.
18. 2 OR 3 OR 4 OR 5
19. 6 OR 7 OR 8 OR 9 OR 10
20. 11 OR 12 OR 13 OR 14 OR 15 OR 16
21. 1 AND 18 AND 19 AND 20
22. 21 NOT 17

Scopus Search Strategy

((((TITLE-ABS-KEY(frail*)) AND (TITLE-ABS-KEY(Prevalence)) OR (TITLE-ABS-KEY(Percent*)) OR (TITLE-ABS-KEY ("were frail")) OR (TITLE-ABS-KEY ("considered frail"))) AND (((TITLE-ABS-KEY(Hospital*)) OR (TITLE-ABS-KEY(Ward)) OR (TITLE-ABS-KEY(Department)) OR (TITLE-ABS-KEY(surg*)) OR (TITLE-ABS-KEY(unit))))) AND ((ALL(Geriatr*)) OR (ALL("older adult*")) OR (ALL(Elder*)) OR (ALL(retire*)) OR (ALL(old)) OR (ALL(older)) OR (ALL(Patient*)))) AND NOT (TITLE-ABS-KEY("community-dwelling"))

Web of Science Search Strategy

1. TS = Frail*
2. TS = Prevalence
3. TS = Percent*
4. TS = "were frail"
5. TS = "considered frail"
6. TS = Hospital*
7. TS = Ward
8. TS = Department
9. TS = Surg*
10. TS = Unit
11. TS = Geriatr*
12. TS = "older adult"
13. TS = Elder*
14. TS = Retir*
15. TS = Old*
16. TS = Patient*
17. TS = "community-dwelling"
18. #2 OR #3 OR #4 OR #5

For peer review only - http://bmjopen.bmj.com/site/about/guidelines.xhtml

19. #6 OR #7 OR #8 OR #9 OR #10
20. #11 OR #12 OR #13 OR #14 OR #15 OR #16
21. #1 AND #18 AND #19 AND #20
22. #21 NOT #17

CINAHL PLUS Search Strategy

1. AB frail*
2. AB prevalence OR AN Percent* OR AB "were frail" OR AB "considered frail"
3. AB Hospital* OR AB Ward OR AB Department OR AB Surg* OR AB Unit
4. AB Geriatr* OR AB "older adult" OR AB Elder* OR AB Retir* OR AB OLD* OR AB Patient*
5. S1 AND S2 AND S3 AND S4

Cochrane Library Search Strategy

1. frail*:ti,ab,kw (Word variations have been searched)
2. prevalence:ti,ab,kw or percent*:ti,ab,kw or "were frail":ti,ab,kw or "considered frail":ti.ab.kw (Word variations have been searched)
3. hospital*:ti,ab,kw or ward:ti,ab,kw or department:ti,ab,kw or surg*:ti,ab,kw or unit:ti,ab,kw (Word variations have been searched)
4. Geriatr*:ti,ab,kw or "older adult":ti,ab,kw or Elder*:ti,ab,kw or Retir*:ti,ab,kw or Old*:ti,ab,kw (Word variations have been searched)
5. Patient*:ti,ab,kw (Word variations have been searched)
6. #4 OR #5
7. #1 AND #2 AND #3 AND #6

For peer review only

Frailty Levels In Geriatric Hospital in-paTients (FLIGHT)
Systematic Review Search Strategy Screening form
PANINI (Physical Activity and Nutritional INfluences in Ageing) project
University of Birmingham
2016 - 2019

UNIVERSITY OF BIRMINGHAM

"The prevalence of frailty amongst geriatric populations within hospital ward settings: A systematic review"

Inclusion criteria - All studies must:

- have a minimum age of ≥ 65 years
- use a clearly defined and validated operational definition for the classification of frailty
- either assess (or attempt to assess) the whole ward, department, unit, hospital, or clinical population, or employ some form of randomised selection of participants
- occur within a hospital setting, in, or including hospital in-patients*
- report the prevalence of frailty or provide sufficient data to allow the calculation of the prevalence of frailty.

* If a study examines a mixed cohort, only data relating to hospital in-patients will be included.

Exclusion criteria:

- studies not written in English
- studies where the sample are non-hospital in-patients (i.e. outpatients, day patients or community-dwelling individuals)

Systematic Review - Screening procedure

1). Import attached RIS file into your reference manager software (preferably EndNote X8.2)

2). Once imported, scan all title and abstracts for eligibility against the inclusion / exclusion criteria above.

3). Move all studies identified as potentially eligible based on title and abstract into a separate group (EndNote), Or folder (RefWorks, Mendeley).

4). Screen full text of identified studies to determine eligibility.

5). Move all eligible studies into separate group / folder.

6). Make note of excluded studies and reasons for their exclusion based on eligibility criteria, in the attached excel file.

5). Compare identified studies.

6). If all reviewers identify the exact same studies, with no discrepancies, this is the end of the initial screening process for the systematic review.

7). If there are differences in the studies identified by different reviewers - discuss until resolution is determined. In the event a unanimous consensus cannot be met by the three reviewers, the majority consensus will be taken, and a note made of this.

For peer review only - http://bmjopen.bmj.com/site/about/guidelines.xhtml

Author	Title	Initial studies included from full text screening (prior to reviewer discussion)		
		Reviewer 1 (PD)	Reviewer 2 (JA)	Reviewer 3 (EA)

For peer review only

Conclusion of discussion	Decision	Response received from author	Outcome

For peer review only

Key

**Unanimous consensus inclusion**	
**Majority consensus inclusion**	
**Minority consensus inclusion / contact author***	*In event where either there is not majority consensus inclusion or exclusion i.e. one reviewer wishes to include and at least one other wishes to seek further information, or two reviewers wish to seek futher information
**Majority consensus exclusion****	** = Reasons for all excluded studies are given in exclusion form
**Unanimous consensus exclusion****	** = Reasons for all excluded studies are given in exclusion form
Unanimous consensus contact author	
? = Contact author***	** = Reasons for all instances of contacting the study author for clarification or futher information to assess eligibility are outlined in contact author form

✓ = Included

x = Excluded

For peer review only

JBI Critical Appraisal Checklist for Studies Reporting Prevalence Data

Reviewer: ______________________ Date: ______________________

Author: ______________________ Year: __________ Record Number: __________

	Yes	No	Unclear	Not applicable
1. Was the sample representative of the target population?	□	□	□	□
2. Were study participants recruited in an appropriate way?	□	□	□	□
3. Was the sample size adequate?	□	□	□	□
4. Were the study subjects and the setting described in detail?	□	□	□	□
5. Was the data analysis conducted with sufficient coverage of the identified sample?	□	□	□	□
6. Were objective, standard criteria used for the measurement of the condition?	□	□	□	□
7. Was the condition measured reliably?	□	□	□	□
8. Was there appropriate statistical analysis?	□	□	□	□
9. Are all important cofounding factors/ subgroups/ differences identified and accounted for?	□	□	□	□
10. Were subpopulations identified using objective criteria?	□	□	□	□

Overall appraisal: Include □ Exclude □ Seek further info □

____

____

____

____

Critical appraisal tool guidelines

Answer: Yes, No, Unclear or Not/Applicable.

1. **Was the sample representative of the target population?**

For peer review only

This question relies upon knowledge of the broader characteristics of the population of interest. If the study is of women with breast cancer, knowledge of at least the characteristics, demographics and medical history is needed. The term "target population" should not be taken to infer every individual from everywhere or with similar disease or exposure characteristics. Instead, give consideration to specific population characteristics in the study, including age range, gender, morbidities, medications, and other potentially influential factors. For example, a sample may not be representative of the target population if a certain group has been used (such as those working for one organisation, or one profession) and the results then inferred to the target population (i.e. working adults).

2. Were study participants recruited in an appropriate way?

Recruitment is the calling or advertising strategy for gaining interest in the study, and is not the same as sampling. Studies may report random sampling from a population, and the methods section should report how sampling was performed. What source of data were study participants recruited from? Was the sampling frame appropriate? For example, census data is a good example of appropriate recruitment as a good census will identify everybody. Was everybody included who should have been included? Were any groups of persons excluded? Was the whole population of interest surveyed? If not, was random sampling from a defined subset of the population employed? Was stratified random sampling with eligibility criteria used to ensure the sample was representative of the population that the researchers were generalizing to?

3. Was the sample size adequate?

An adequate sample size is important to ensure good precision of the final estimate. Ideally we are looking for evidence that the authors conducted a sample size calculation to determine an adequate sample size. This will estimate how many subjects are needed to produce a reliable estimate of the measure(s) of interest. For conditions with a low prevalence, a larger sample size is needed. Also consider sample sizes for subgroup (or characteristics) analyses, and whether these are appropriate. Sometimes, the study will be large enough (as in large national surveys) whereby a sample size calculation is not required. In these cases, sample size can be considered adequate.

When there is no sample size calculation and it is not a large national survey, the reviewers may consider conducting their own sample size analysis using the following formula:[15, 16]

N = Z2P(1-P)

d2

Where:

- N = sample size
- Z = Z statistic for a level of confidence
- P = Expected prevalence or proportion (in proportion of one; if 20%, P = 0.2)
- d = precision (in proportion of one; if 5%, d=0.05)

4. Were the study subjects and setting described in detail?

Certain diseases or conditions vary in prevalence across different geographic regions and populations (e.g. women vs. men, sociodemographic variables between countries). Has the study sample been described in sufficient detail so that other researchers can determine if it is comparable to the population of interest to them?

5. Is the data analysis conducted with sufficient coverage of the identified sample?

A large number of dropouts, refusals or "not founds" amongst selected subjects may diminish a study's validity, as can low response rates for survey studies.

- Did the authors describe the reasons for non-response and compare persons in the study to those not in the study, particularly with regards to their socio-demographic characteristics?

- Could the not-responders have led to an underestimate of prevalence of the disease or condition under investigation?

- If reasons for non-response appear to be unrelated to the outcome measured and the characteristics of non-responders are comparable to those in the study, the researchers may be able to justify a more modest response rate.

- Did the means of assessment or measurement negatively affect the response rate (measurement should be easily accessible, conveniently timed for participants, acceptable in length and suitable in content).

6. Were objective, standard criteria used for measurement of the condition?

Here we are looking for measurement or classification bias. Many health problems are not easily diagnosed or defined, and some measures may not be capable of including or excluding appropriate levels or stages of the health problem. If the outcomes were assessed based on existing definitions or diagnostic criteria, then the answer to this question is likely to be yes. If the outcomes were assessed using observer reported, or self-reported scales, the risk of over-or under-reporting is increased, and objectivity is compromised. Importantly, determine if the measurement tools used were validated instruments as this has a significant impact on outcome assessment validity.

7. Was the condition measured reliably?

Considerable judgment is required to determine the presence of some health outcomes. Having established the objectivity of the outcome measurement instrument (see item 6 of this scale), it is important to establish how the measurement was conducted. Were those involved in collecting data trained or educated in the use of the instrument/s? If there was more than one data collector, were they similar in terms of level of education, clinical or research experience, or level of responsibility in the piece of research being appraised?

- Has the researcher justified the methods chosen?
- Has the researcher made the methods explicit? (For interview method, how were interviews conducted?)

8. Was there appropriate statistical analysis?

As with any consideration of statistical analysis, consideration should be given to whether there was a more appropriate alternate statistical method that could have been used. The methods section should be detailed enough for reviewers to identify the analytical technique used and how specific variables were measured. Additionally, it is also important to assess the appropriateness of the analytical strategy in terms of the assumptions associated with the approach as differing methods of analysis are based on differing assumptions about the data and how it will respond. Prevalence rates found in studies only provide estimates of the true prevalence of a problem in the larger population. Since some subgroups are very small, 95% confidence intervals are usually given.

9. Are all important confounding factors/ subgroups/differences identified and accounted for?

Incidence and prevalence studies often draw or report findings regarding the differences between groups. It is important that authors of these studies identify all important confounding factors, subgroups and differences and account for these.

10. Were subpopulations identified using objective criteria?

Objective criteria should also be used where possible to identify subgroups (refer to question 6).

If a study scores less than 5/10 (50%) it should be excluded, unless there is ambiguity with relation to the aforementioned criteria, in which case more information should be sought and then the criteria rereviewed.

For peer review only

Study	Study details					Setting
	Author	Year of Publication	Study title	Journal of publication	Aim	
Study 1						
Study 2						
Study 3						
Study 4						
Study 5						
Study 6						
Study 7						
Study 8						
Study 9						
Study 10						
Study 11						
Study 12						
Study 13						
Study 14						
Study 15						
Study 16						
Study 17						
Study 18						
Study 19						
Study 20						
Study 21						
Study 22						
Study 23						
Study 24						
Study 25						
Study 26						
Study 27						
Study 28						
Study 29						
Study 30						
Study 31						
Study 32						
Study 33						
Study 34						
Study 35						
Study 36						
Study 37						
Study 38						
Study 39						
Study 40						
Study 41						
Study 42						
Study 43						
Study 44						
Study 45						
Study 46						
Study 47						
Study 48						
Study 49						

For peer review only

Study 50
Study 51
Study 52
Study 53
Study 54
Study 55
Study 56
Study 57
Study 58
Study 59
Study 60
Study 61
Study 62
Study 63
Study 64
Study 65
Study 66
Study 67
Study 68
Study 69
Study 70
Study 71
Study 72
Study 73
Study 74
Study 75
Study 76
Study 77
Study 78
Study 79
Study 80
Study 81
Study 82
Study 83
Study 84
Study 85
Study 86
Study 87
Study 88
Study 89
Study 90
Study 91
Study 92
Study 93
Study 94
Study 95
Study 96
Study 97
Study 98
Study 99
Study 100
Study 101
Study 102

For peer review only

Study 103
Study 104
Study 105
Study 106
Study 107
Study 108
Study 109
Study 110
Study 111
Study 112
Study 113
Study 114
Study 115

For peer review only

Ward / Department / unit / hospital / clinical population type	Study design	Study duration	Age of participants (mean +/-SD)	Age of participants (range)

For peer review only

For peer review only

For peer review only

Study Methods

	Subject characteristics	
Sex (proportion male / female participants)	Country / location	Sample size (n)

For peer review only

For peer review only

For peer review only

Diagnosis / Prevalent morbidity (if applicable)	Any other relevant characteristic

For peer review only

For peer review only

For peer review only

Criteria utilised for the operational definition of frailty	Dependent variables

For peer review only

For peer review only

For peer review only

		Results
Methods of data analysis	Prevalence of frailty	Prevalence of "pre-frailty"

For peer review only

For peer review only

For peer review only

	Authors comments	Reviewers comments
Prevalence of robust/non frail		

For peer review only

For peer review only

For peer review only

Planned stratified analysis

For peer review only

Study 1

Study details	Authors	Year of Publication	Study title	Journal of publication	Aim

For peer review only

PRISMA-P (Preferred Reporting Items for Systematic review and Meta-Analysis Protocols) 2015 checklist: recommended items to address in a systematic review protocol*

Section and topic	Item No	Checklist item	Page no.
**ADMINISTRATIVE INFORMATION**			
Title:			
Identification	1a	Identify the report as a protocol of a systematic review	1
Update	1b	If the protocol is for an update of a previous systematic review, identify as such	
Registration	2	If registered, provide the name of the registry (such as PROSPERO) and registration number	2
Authors:			
Contact	3a	Provide name, institutional affiliation, e-mail address of all protocol authors; provide physical mailing address of corresponding author	1
Contributions	3b	Describe contributions of protocol authors and identify the guarantor of the review	1,9
Amendments	4	If the protocol represents an amendment of a previously completed or published protocol, identify as such and list changes; otherwise, state plan for documenting important protocol amendments	
Support:			
Sources	5a	Indicate sources of financial or other support for the review	8,9
Sponsor	5b	Provide name for the review funder and/or sponsor	8,9
Role of sponsor or funder	5c	Describe roles of funder(s), sponsor(s), and/or institution(s), if any, in developing the protocol	
**INTRODUCTION**			
Rationale	6	Describe the rationale for the review in the context of what is already known	3
Objectives	7	Provide an explicit statement of the question(s) the review will address with reference to participants, interventions, comparators, and outcomes (PICO)	4
**METHODS**			
Eligibility criteria	8	Specify the study characteristics (such as PICO, study design, setting, time frame) and report characteristics (such as years considered, language, publication status) to be used as criteria for eligibility for the review	4
Information sources	9	Describe all intended information sources (such as electronic databases, contact with study authors, trial registers or other grey literature sources) with planned dates of coverage	4,5,6
Search strategy	10	Present draft of search strategy to be used for at least one electronic database, including planned limits, such that it could be repeated	Appendix 1
Study records:			

For peer review only

Data management	11a	Describe the mechanism(s) that will be used to manage records and data throughout the review	5,6
Selection process	11b	State the process that will be used for selecting studies (such as two independent reviewers) through each phase of the review (that is, screening, eligibility and inclusion in meta-analysis)	5,6,7,8
Data collection process	11c	Describe planned method of extracting data from reports (such as piloting forms, done independently, in duplicate), any processes for obtaining and confirming data from investigators	7
Data items	12	List and define all variables for which data will be sought (such as PICO items, funding sources), any pre-planned data assumptions and simplifications	7
Outcomes and prioritization	13	List and define all outcomes for which data will be sought, including prioritization of main and additional outcomes, with rationale	7
Risk of bias in individual studies	14	Describe anticipated methods for assessing risk of bias of individual studies, including whether this will be done at the outcome or study level, or both; state how this information will be used in data synthesis	6
Data synthesis	15a	Describe criteria under which study data will be quantitatively synthesised	7,8
	15b	If data are appropriate for quantitative synthesis, describe planned summary measures, methods of handling data and methods of combining data from studies, including any planned exploration of consistency (such as I^2, Kendall's τ)	7,8
	15c	Describe any proposed additional analyses (such as sensitivity or subgroup analyses, meta-regression)	8
	15d	If quantitative synthesis is not appropriate, describe the type of summary planned	8
Meta-bias(es)	16	Specify any planned assessment of meta-bias(es) (such as publication bias across studies, selective reporting within studies)	
Confidence in cumulative evidence	17	Describe how the strength of the body of evidence will be assessed (such as GRADE)	

*** It is strongly recommended that this checklist be read in conjunction with the PRISMA-P Explanation and Elaboration (cite when available) for important clarification on the items. Amendments to a review protocol should be tracked and dated. The copyright for PRISMA-P (including checklist) is held by the PRISMA-P Group and is distributed under a Creative Commons Attribution Licence 4.0.**

From: Shamseer L, Moher D, Clarke M, Ghersi D, Liberati A, Petticrew M, Shekelle P, Stewart L, PRISMA-P Group. Preferred reporting items for systematic review and meta-analysis protocols (PRISMA-P) 2015: elaboration and explanation. BMJ. 2015 Jan 2;349(jan02 1):g7647.

For peer review only

BMJ Open

Frailty Levels in Geriatric Hospital paTients (FLIGHT) - The prevalence of frailty amongst geriatric populations within hospital ward settings: A systematic review protocol

Journal:	*BMJ Open*
Manuscript ID	bmjopen-2019-030147.R1
Article Type:	Protocol
Date Submitted by the Author:	20-May-2019
Complete List of Authors:	Doody, Paul; University of Birmingham, School of Sport, Exercise and Rehabilitation Sciences<br>Aunger, Justin; University of Birmingham, School of Sport, Exercise and Rehabilitation Sciences<br>Asamane, Evans; University of Birmingham, School of Sport, Exercise and Rehabilitation Sciences<br>Greig, Carolyn; School of Sport, Exercise and Rehabilitation Sciences, University of Birmingham, Edgbaston, Birmingham, B15 2TT, UK; MRC-Arthritis Research UK Centre for Musculoskeletal Ageing Research<br>Lord, Janet; University of Birmingham, NIHR Surgical Reconstruction & Microbiology Research Centre<br>Whittaker, Anna; University of Birmingham, School of Sport, Exercise and Rehabilitation Sciences; University of Stirling
**Primary Subject Heading**:	Geriatric medicine
Secondary Subject Heading:	Geriatric medicine, Health economics
Keywords:	Frail, Geriatric, inpatient, older adult, prevalence, systematic review

SCHOLARONE™
Manuscripts

Frailty Levels in Geriatric Hospital paTients (FLIGHT) - The prevalence of frailty amongst geriatric populations within hospital ward settings: A systematic review protocol

Paul Doody[1], Justin A. Aunger[1], Evans A. Asamane[1], Carolyn A. Greig[1,2,3], Janet M. Lord[2], Anna C. Whittaker[1,4]

[1] School of Sport, Exercise and Rehabilitation Sciences, University of Birmingham, United Kingdom

[2] Institute of Inflammation and Ageing MRC-ARUK Centre for Musculoskeletal Ageing Research, University of Birmingham, United Kingdom

[3] NIHR Birmingham Biomedical Research Centre, University Hospitals Birmingham NHS Foundation Trust and University of Birmingham, United Kingdom

[4] Faculty of Health Sciences and Sport, University of Stirling, United Kingdom

Corresponding author: Paul Doody (p.d.doody@bham.ac.uk, +44 (0) 121 414 4125), School of Sport Exercise and Rehabilitation Sciences (G35), University of Birmingham, United Kingdom

For peer review only

Abstract

Introduction: Frailty is a common and clinically significant condition in geriatric populations, associated with adverse health outcomes such as hospitalisation, disability, and mortality. Although there are systematic reviews/meta-analyses assessing the prevalence of frailty in community-dwelling older adults, nursing home residents, and cancer and general surgery patients, there are none assessing the overall prevalence of frailty in geriatric hospital inpatients.

Methods and analysis: This review will systematically search and analyse the prevalence of frailty within geriatric hospital inpatients within the literature. A search will be employed on the platforms of Ovid, Web of Science, and databases of CINAHL Plus, SCOPUS, and the Cochrane Library. Any observational or experimental study design which utilises a validated operational definition of frailty, reports the prevalence of frailty, has a minimum age ≥ 65 years, attempts to assess the whole ward/clinical population, and occurs in hospital inpatients, will be included. Title and abstract and full-text screenings will be conducted by three reviewers. Methodological quality of eligible studies will be assessed utilising the Joanna Briggs Institute critical appraisal tool. Data extraction will be performed by two reviewers. If sufficient data are available, a meta-analysis synthesising pooled estimates of the prevalence of frailty and pre-frailty, as well as the prevalence of frailty stratified by age, sex, operational frailty definition, prevalent morbidities, ward type, and location, among older hospitalised in-patients will be conducted. Clinical heterogeneity will be assessed by two reviewers. Statistical heterogeneity will be assessed through a Cochran Q test, and an I^2 test performed to assess its magnitude.

Ethics and dissemination: Ethical approval was not required as primary data will not be collected. Findings will be disseminated through publication in peer reviewed open access scientific journals, public engagement events, conference presentations, and social media**.**

Trial Registration number: This study has been registered on PROSPERO (registration number 79202).

Strengths and limitations of this study:

For peer review only - http://bmjopen.bmj.com/site/about/guidelines.xhtml

- First review to systematically or exclusively assess the overall prevalence of frailty in geriatric hospital inpatients
- Will seek to provide stratified analysis of the prevalence of frailty based on age, sex, operational frailty definition, prevalent morbidities, ward type and location
- Three independent reviewers during screening phase; ensuring high internal reliability and consistency of included studies
- Will include only studies for which the full text is available in English, therefore will likely be relatively over-representative of Western nations (Europe, Australasia, and the Americas); although this is true of scientific publications in general.
- **Keywords**: department; frail; geriatric; hospital; inpatient; meta-analysis; older adult; prevalence; systematic review; ward.

For peer review only

Introduction

Frailty is a common and clinically significant condition within geriatric populations (1), predominantly due to its association with adverse health outcomes such as hospitalization, disability and mortality (1-6). Although there are systematic reviews and meta-analysis assessing the prevalence of frailty amongst community-dwelling older adults (7-10), nursing home residents (11), and cancer (12) and general surgery patients (13), presently there are no systematic reviews or meta-analysis which assess the overall prevalence of frailty among geriatric hospital inpatients. This constitutes an important gap in the literature which needs to be addressed and has important consequences. Such consequences include the tailoring of services within this setting to the needs of service users, for example, the potential implementation of exercise rehabilitation treatments within this setting for this cohort; with physical activity and exercise being proposed as potentially offering the best form of treatment for frail older adults (14), and shown to be capable of reducing, and even reversing frailty within older adults (15,16). Through providing a highly detailed analysis of the prevalence of frailty amongst older population within this setting, this review has the potential to aid in the facilitation of improvements in the planning and orientation of organisational structures and resources, to meet the needs of this population, and enhance the care of frail older adults in inpatient hospital settings.

Methods and Design

Review Aim:

The aim of this review is to systematically search and analyse the prevalence of frailty amongst geriatric populations (aged ≥ 65 years) within inpatient hospital settings within the literature. If a meta-analysis proves possible, the aim of this study is also to synthesise pooled estimates of the prevalence of frailty and pre-frailty, as well as the prevalence of frailty stratified by age, sex, operational frailty definition, prevalent morbidities, ward type and location, among hospital in-patients.

For peer review only - http://bmjopen.bmj.com/site/about/guidelines.xhtml

Review Objectives:

1) To identify and compare studies reporting the prevalence of frailty within hospital ward settings.
2) To combine the extracted data to calculate the pooled overall prevalence of frailty in hospitalised geriatric in-patients.
3) To perform stratified analysis of the prevalence of frailty based on age, sex, operational frailty definition, prevalent morbidity and ward type in order to assess the relationship between frailty and these factors.

Eligibility criteria:

Inclusion criteria: all studies must have a minimum age of ≥ 65 years, use a clearly defined and validated operational definition for the classification of frailty (i.e. has been specifically validated for the assessment of frailty, either through comparison with existing validated tools, or its predictive value regarding negative health outcomes aligned with frailty), either assess (or attempt to assess) the whole ward, department, unit, hospital or specific clinical population, or employ some form of randomised selection of participants, occur within a hospital setting, in, or including, hospital in-patients (operationally defined as any patient admitted to hospital who remains overnight, or were initially expected to remain overnight), report the prevalence of frailty or provide sufficient data to allow the calculation of the prevalence of frailty. If a study examines a mixed cohort, only data relating to hospital in-patients will be included in the review.

Exclusion criteria: all studies not written in English, studies where the sample are not hospital inpatients (i.e. outpatients, day patients or community-dwelling individuals).

Information sources:

Searches will be conducted on the platforms of Ovid (incorporating the databases of Journals @Ovid full text, EMBASE, CAB abstracts, Ovid MEDLINE ® In process and other non-indexed citations, Ovid MEDLINE ®, and PyschINFO) and Web of Science (incorporating the databases of Science Citation Index Expanded (SCI-Expanded), Conference Proceedings Citation Index – Science (CRI-S), and Emerging

For peer review only - http://bmjopen.bmj.com/site/about/guidelines.xhtml

For peer review only

Sources Citation Index (ESCI)), and the databases of CINAHL Plus, SCOPUS, and the Cochrane Library databases (the Cochrane Database of Systematic Reviews (CDSR), the Cochrane Central Register of Controlled Trials (CENTRAL), the Cochrane Methodology Register (CMR), the Database of Abstracts of Reviews of Effect (DARE), Health Technology Assessment database (HTA) and the NHS Economic Evaluation Database (EED)).

Types of studies:

Any form of observational or experimental study design which assesses the prevalence of frailty and meets the above eligibility criteria. For longitudinal observational studies, and experimental studies, frailty scores and additional data will be extracted from baseline data, provided baseline data meets the above eligibility criteria.

Search Strategy:

The search strategy will be conducted on the two platforms of Ovid and Web of Science, as well as the databases of SCOPUS, CINAHL Plus, and the Cochrane Library databases (Appendix 1). These searches will encompass all available literature published prior to 21/11/2018.

Screening:

Prior to the commencement of title and abstract screening by the three independent reviewers, duplicates will be removed utilising EndNote X8.2. The reduced list of studies will be manually screened for the removal of any remaining duplicates. All reviewers will be provided with an instructional screening form (Appendix 2), and a .ris file containing all studies captured within database searches. The screening form will list the eligibility criteria and instructions on setting up the .ris file for screening within a reference manager.

For peer review only - http://bmjopen.bmj.com/site/about/guidelines.xhtml

The title and abstract of all studies will then be independently screened by the three reviewers, with each reviewer placing potentially eligible studies into a separate folder. Upon completion, potentially eligible studies from all three reviewers will be placed into a "master folder" and the results collated. Duplicates will be removed, leaving the final combined list of studies for the full text screening phase. All reviewers will then independently screen the full text of remaining studies utilising the screening form and maintain separate files for included and excluded studies (including reasons), as well as for studies for which the reviewer feels the need to contact the authors for clarification or additional information.

Upon completion, a full text screening master file (Appendix 3) will be formulated by the lead reviewer displaying each reviewer's full text screening decision for each study. All three reviewers will then meet to discuss the decisions of each study and endeavour to come to an agreement on studies for which there is not initial unanimous consensus. During this process a full list of included and excluded studies (with reasons), and studies for which reviewers agree to contact authors for additional information or clarification will be formed by the lead reviewer. The lead reviewer will then contact study authors and, upon receipt of clarification or additional information, will meet with reviewers to discuss the inclusion/exclusion of the study.

Manual screening will also be employed by reviewers and include the reference lists of all included studies, as well as excluded but potentially relevant studies or systematic reviews captured within the screening. As part of the grey literature search of this review, in process publications will also be searched and conference abstracts will be followed up with authors to ascertain if a full text relating to the data is available. Studies of the same cohort will be included only once, using the study which provides the most information about the cohort relevant to this review.

Assessment of methodological quality:

The quality of eligible studies from full text screening will be assessed by two reviewers independently using the Joanna Briggs Institute (JBI) critical appraisal tool for studies reporting prevalence data (17)

For peer review only - http://bmjopen.bmj.com/site/about/guidelines.xhtml

(Appendix 4). In the event of any discrepancies between the two reviewers, a consensus will be attempted to be reached by discussion. In the event a full consensus cannot be reached between the two reviewers after an exhaustive discussion, the opinion of a third reviewer will be obtained, and the proceeding majority consensus will be taken.

Data extraction:

Data extraction will be performed by two reviewers independently. In the event of any discrepancies between the two reviewers, a consensus will be attempted to be reached by discussion. In the event that a full consensus cannot be reached between the two reviewers after an exhaustive discussion, the opinion of a third reviewer will be obtained, and the proceeding majority consensus will be taken.

The following data, where available, will be extracted from all eligible studies (see Appendix 5 for template). If any data are not immediately available, the authors of the studies in question will be contacted in an attempt to retrieve all applicable data:

Study details: authors, year of publication, study title, journal of publication, aim. Study methods: setting, ward/department/unit/hospital type/clinical population, study design, recruitment duration, subject characteristics (age of participants (mean and standard deviation, range)), sex (proportion of male / female participants), country / continent, sample size, diagnosis / prevalent morbidity (if applicable), any other relevant characteristics), criteria utilised for the operational definition of frailty. Results: Number of frail participants, number of "pre-frail" participants, number of robust / non-frail participants, prevalence of frailty, prevalence of pre-frailty, prevalence of robustness / non-frailty, number of male participants, number of frail male participants, number of pre-frail male participants, number of non-frail / robust male participants, prevalence of frailty in male participants, prevalence of pre-frailty in male participants, prevalence of non-frailty / robustness in male participants, number of female participants, number of frail female participants, number of pre-frail female participants, number of non-frail / robust female participants, prevalence of frailty in female participants, prevalence of pre-frailty in female participants, prevalence of non-frailty / robustness in female participants, and finally authors comments and reviewers' comments.

For peer review only

External to the studies, data will also be extracted with regard to the 5 year average Gross Domestic Product (GDP) per capita Purchasing Power Parity (PPP) (current international $) of the country in which each study takes place, incorporating the five years directly preceding the commencement of recruitment to the study (18). External data will also be extracted with regard to the 5 year average health care expenditure per capita PPP (current international $) of the country in which each study takes place, incorporating the five years directly preceding the commencement of recruitment to the study (19). Each calendar year of the study will also be included provided recruitment continues through to > 6 months in the preceding year.

Data synthesis:

Quantitative synthesis (Meta-Analysis): If a sufficient quantity of identified studies are comparable, a meta-analysis, pooling the aggregated data from each study, will be performed. Clinical heterogeneity will be assessed by two reviewers based on their judgement of the available data and any disagreements will be discussed thoroughly with the aim of reaching a unanimous consensus. If a unanimous consensus cannot be reached, the opinion of a third reviewer will be sought, and the proceeding majority consensus will be taken. Statistical heterogeneity will be assessed through the utilisation of a Cochran Q test and considered present at $p < .05$. An I^2 test will be performed in order to assess the magnitude of this heterogeneity, with I^2 values of 25%, 50% and 75% being considered low, moderate and high respectively. If the Cochrane Q statistic test detected statistically significant heterogeneity, combined with the researcher's assessment, a randomised-effects model will be utilised. Given the nature of this review and in particular its overall aim, combined with the eligible studies identified in preliminary searches, it is likely the initial quantitative synthesis will utilise a random-effects model.

Stratified analysis will also be conducted according to age (65 – 74 years, 75 – 84 years and 85+ years), sex, operational frailty definition, ward type, prevalent morbidity and location where possible. These variables have been specifically chosen for stratified analysis predominantly due to an enhanced knowledge of these areas being of practical utility to researchers and clinicians; stemming from empirical evidence persistently showing alterations in these factors to impact upon the prevalence of frailty (2,4,20-22). As such stratified

For peer review only

analysis pertaining to these variables will facilitate this review to provide a more in-depth and thorough insight into the prevalence of frailty amongst geriatric hospital inpatients.

Clinical heterogeneity for stratified analysis will be assessed by two reviewers based on their judgement of the available data. Any disagreements will be discussed thoroughly with the aim of reaching a unanimous consensus. If a unanimous consensus cannot be reached, the opinion of a third reviewer will be sought. Statistical heterogeneity for sub-analysis will similarly be assessed through the utilisation of a Cochran Q test and considered present at $p < .05$. An I^2 test will be performed in order to assess the magnitude of this heterogeneity, with I^2 values of 25%, 50% and 75% being considered low, moderate and high respectively.

Similarly, it is likely a random-effects model will be utilised to synthesise pooled estimates of the prevalence of frailty stratified by these criteria (although there is more of a likelihood that a fixed effects model could potentially be utilised within these analyses, in comparison to the initial analysis, given the nature of stratified analysis).

Correlation analysis will also be employed to examine the relationship between the prevalence of frailty of geriatric inpatients and economic prosperity (GDP per capita PPP) (current international $), and health care expenditure (per capita PPP) (current international $). Additionally, multi-linear regression analysis will examine the predictive value between economic prosperity and health care expenditure, and the prevalence of frailty of geriatric inpatients.

Qualitative synthesis: if a meta-analysis is not possible based on the nature of the studies and the data available, a more thorough systematic narrative analysis will be conducted, with findings presented in both textual and tabular formats.

Patient and Public involvement

All authors are strong proponents of patient and public involvement and engagement with research and believe the finding of this review will be important to aid the facilitation of improvements in the planning

and orientation of organisation structures and resources within this setting to meet the needs of service users; specifically relating to the enhanced care of older adults in inpatient hospital settings. However, given the nature of this study (systematic review), it was not possible to involve the public. However, the findings will be disseminated to our patient and public involvement groups.

Ethics and Dissemination

Formal ethical approval was not required for this review as primary data will not be collected. The findings of this study will be disseminated through publication in the form of scientific papers in peer reviewed open access scientific journals, public engagement events within the United Kingdom and Europe, online via social media (Twitter, Instagram) and the PANINI project website (23,24), and presentation at conferences within the UK and internationally.

Funding

This review has been funded by the European Commission's Horizon 2020 research and innovation programme under the Marie Sklodowska-Curie grant agreement (675003); of which PD, EA and JA are Marie Sklodowska-Curie Doctoral Research Fellows, AW, JL and CG doctoral supervisors, and AW the grants Principal Investigator.

Author contributions

PD is guarantor and lead reviewer. PD designed the systematic review protocol, conducted the literature searches and prepared this manuscript for publication, with supervision, input and feedback from AW, CG and JL. EA and JA are independent reviewers for title and abstract and full text screenings. JA will also act as independent data extractor for included studies. All authors have read and approved the final manuscript.

For peer review only

Conflicts of interest:

The authors of this review report no known conflicts of interest.

Acknowledgements:

The authors of this review would like to thank and acknowledge Ms. Lynne Harris (Subject advisor of the Main Library at the University of Birmingham, United Kingdom) for her assistance during the formulation of the search strategy utilised within this systematic review.

Word count (excluding title page, abstract, author contributions, acknowledgements and references): 2507

For peer review only

References:

(1) Rodriguez-Mañas L, Fried LP. Frailty in the clinical scenario. The Lancet 2015 /02/14;385(9968):e9.

(2) Fried LP, Tangen CM, Walston J, Newman AB, Hirsch C, Gottdiener J, et al. Frailty in older adults evidence for a phenotype. The Journals of Gerontology Series A: Biological Sciences and Medical Sciences 2001;56(3):M157.

(3) Gill TM, Gahbauer EA, Allore HG, Han L. Transitions between frailty states among community-living older persons. Arch Intern Med 2006;166(4):418-423.

(4) Sternberg SA, Schwartz AW, Karunananthan S, Bergman H, Mark Clarfield A. The identification of frailty: a systematic literature review. J Am Geriatr Soc 2011;59(11):2129-2138.

(5) Clegg A, Young J, Iliffe S, Rikkert MO, Rockwood K. Frailty in elderly people. The Lancet 2013 March 8,;381(9868):752-762.

(6) Sourial N, Bergman H, Karunananthan S, Wolfson C, Payette H, Gutierrez-Robledo LM, et al. Implementing Frailty Into Clinical Practice: A Cautionary Tale. J Gerontol A Biol Sci Med Sci 2013 -05-02:glt053.

(7) Collard RM, Boter H, Schoevers RA, Oude Voshaar RC. Prevalence of frailty in community-dwelling older persons: a systematic review. J Am Geriatr Soc 2012 Aug;60(8):1487-1492.

(8) Siriwardhana DD, Hardoon S, Rait G, Weerasinghe MC, Walters KR. Prevalence of frailty and prefrailty among community-dwelling older adults in low-income and middle-income countries: a systematic review and meta-analysis. BMJ open 2018;8(3):e018195.

(9) Kojima G, Iliffe S, Taniguchi Y, Shimada H, Rakugi H, Walters K. Prevalence of frailty in Japan: A systematic review and meta-analysis. Journal of epidemiology 2017;27(8):347-353.

(10) Verlaan S, Ligthart-Melis GC, Wijers SL, Cederholm T, Maier AB, de van der Schueren, Marian AE. High prevalence of physical frailty among community-dwelling malnourished older adults–a systematic review and meta-analysis. Journal of the American Medical Directors Association 2017;18(5):374-382.

(11) Kojima G. Prevalence of frailty in nursing homes: A systematic review and meta-analysis. Journal of the American Medical Directors Association 2015;16(11):940-945.

(12) Handforth C, Clegg A, Young C, Simpkins S, Seymour MT, Selby PJ, et al. The prevalence and outcomes of frailty in older cancer patients: a systematic review. Annals of Oncology 2015 June;26(6):1091-1101.

(13) Hewitt J, Long S, Carter B, Bach S, McCarthy K, Clegg A. The prevalence of frailty and its association with clinical outcomes in general surgery: a systematic review and meta-analysis. Age Ageing 2018;47(6):793-800.

(14) Theou O, Stathokostas L, Roland KP, Jakobi JM, Patterson C, Vandervoort AA, et al. The effectiveness of exercise interventions for the management of frailty: a systematic review. Journal of aging research 2011;2011.

(15) Fiatarone MA, O'Neill EF, Ryan ND, Clements KM, Solares GR, Nelson ME, et al. Exercise training and nutritional supplementation for physical frailty in very elderly people. N Engl J Med 1994;330(25):1769-1775.

(16) Tarazona-Santabalbina FJ, Gmez-Cabrera MC, Prez-Ros P, Martnez-Arnau FM, Cabo H, Tsaparas K, et al. A multicomponent exercise intervention that reverses frailty and improves cognition, emotion, and social networking in the community-dwelling frail elderly: a randomized clinical trial. Journal of the American Medical Directors Association 2016;17(5):426-433.

For peer review only

(17) Munn Z, Moola S, Riitano D, Lisy K. The development of a critical appraisal tool for use in systematic reviews addressing questions of prevalence. International journal of health policy and management 2014;3(3):123.

(18) International Monetary Fund (. World Economic Outlook (April 2019). 2019; Available at: https://www.imf.org/external/datamapper/PPPPC@WEO/ADVEC/WEOWORLD. Accessed 02/05/, 2019.

(19) World Health Organisation (. Global Health Expenditure Database (GHED). 2019; Available at: http://apps.who.int/nha/database/Home/Index/en. Accessed 02/05/, 2019.

(20) Purser JL, Kuchibhatla MN, Fillenbaum GG, Harding T, Peterson ED, Alexander KP. Identifying frailty in hospitalized older adults with significant coronary artery disease. J Am Geriatr Soc 2006;54(11):1674-1681.

(21) Santos-Eggimann B, Cuénoud P, Spagnoli J, Junod J. Prevalence of frailty in middle-aged and older community-dwelling Europeans living in 10 countries. The Journals of Gerontology: Series A 2009;64(6):675-681.

(22) Andela RM, Dijkstra A, Slaets JPJ, Sanderman R. Prevalence of frailty on clinical wards: Description and implications; 20158543. Int J Nurs Pract 2010;16(1):14-19.

(23) Whittaker AC, Delledonne M, Finni T, Garagnani P, Greig C, Kallen V, et al. Physical Activity and Nutrition INfluences In ageing (PANINI): consortium mission statement. Aging clinical and experimental research 2018:1-8.

(24) University of Birmingham. Physical Activity and Nutritional INfluences In ageing (PANINI) Available at: http://www.birmingham.ac.uk/generic/panini/index.aspx. Accessed 6th February, 2019.

For peer review only

Search Strategy:

Ovid Search Strategy

1. Frail$.ti.ab.
2. Prevalence.ti,ab.
3. Percent$.ti,ab.
4. “were frail”.ti,ab.
5. “considered frail”.ti,ab.
6. Hospital$.ti,ab.
7. Ward.ti,ab.
8. Department.ti,ab.
9. Surg*.ti,ab.
10. Unit.ti,ab.
11. Geriatr*.tx.
12. “older adult*”.tx.
13. Elder$.tx.
14. Retire*.tx.
15. Old$.tx.
16. Patient$.tx.
17. “community-dwelling”.ti,ab.
18. 2 OR 3 OR 4 OR 5
19. 6 OR 7 OR 8 OR 9 OR 10
20. 11 OR 12 OR 13 OR 14 OR 15 OR 16
21. 1 AND 18 AND 19 AND 20
22. 21 NOT 17

Scopus Search Strategy

((((TITLE-ABS-KEY(frail*)) AND (TITLE-ABS-KEY(Prevalence)) OR (TITLE-ABS-KEY(Percent*)) OR (TITLE-ABS-KEY ("were frail")) OR (TITLE-ABS-KEY ("considered frail"))) AND (((TITLE-ABS-KEY(Hospital*)) OR (TITLE-ABS-KEY(Ward)) OR (TITLE-ABS-KEY(Department)) OR (TITLE-ABS-KEY(surg*)) OR (TITLE-ABS-KEY(unit))))) AND ((ALL(Geriatr*)) OR (ALL("older adult*")) OR (ALL(Elder*)) OR (ALL(retire*)) OR (ALL(old)) OR (ALL(older)) OR (ALL(Patient*)))) AND NOT (TITLE-ABS-KEY("community-dwelling"))

Web of Science Search Strategy

1. TS = Frail*
2. TS = Prevalence
3. TS = Percent*
4. TS = “were frail”
5. TS = “considered frail”
6. TS = Hospital*
7. TS = Ward
8. TS = Department
9. TS = Surg*
10. TS = Unit
11. TS = Geriatr*
12. TS = “older adult”
13. TS = Elder*
14. TS = Retir*
15. TS = Old*
16. TS = Patient*
17. TS = “community-dwelling”
18. #2 OR #3 OR #4 OR #5

19. #6 OR #7 OR #8 OR #9 OR #10
20. #11 OR #12 OR #13 OR #14 OR #15 OR #16
21. #1 AND #18 AND #19 AND #20
22. #21 NOT #17

CINAHL PLUS Search Strategy

1. AB frail*
2. AB prevalence OR AN Percent* OR AB "were frail" OR AB "considered frail"
3. AB Hospital* OR AB Ward OR AB Department OR AB Surg* OR AB Unit
4. AB Geriatr* OR AB "older adult" OR AB Elder* OR AB Retir* OR AB OLD* OR AB Patient*
5. S1 AND S2 AND S3 AND S4

Cochrane Library Search Strategy

1. frail*:ti,ab,kw (Word variations have been searched)
2. prevalence:ti,ab,kw or percent*:ti,ab,kw or "were frail":ti,ab,kw or "considered frail":ti.ab.kw (Word variations have been searched)
3. hospital*:ti,ab,kw or ward:ti,ab,kw or department:ti,ab,kw or surg*:ti,ab,kw or unit:ti,ab,kw (Word variations have been searched)
4. Geriatr*:ti,ab,kw or "older adult":ti,ab,kw or Elder*:ti,ab,kw or Retir*:ti,ab,kw or Old*:ti,ab,kw (Word variations have been searched)
5. Patient*:ti,ab,kw (Word variations have been searched)
6. #4 OR #5
7. #1 AND #2 AND #3 AND #6

For peer review only

Frailty Levels In Geriatric Hospital in-paTients (FLIGHT)
Systematic Review Search Strategy Screening form
PANINI (Physical Activity and Nutritional INfluences in Ageing) project
University of Birmingham
2016 - 2019

UNIVERSITY OF BIRMINGHAM

"The prevalence of frailty amongst geriatric populations within hospital ward settings: A systematic review"

Inclusion criteria - All studies must:

- have a minimum age of ≥ 65 years
- use a clearly defined and validated operational definition for the classification of frailty
- either assess (or attempt to assess) the whole ward, department, unit, hospital, or clinical population, or employ some form of randomised selection of participants
- occur within a hospital setting, in, or including hospital in-patients*
- report the prevalence of frailty or provide sufficient data to allow the calculation of the prevalence of frailty.

* If a study examines a mixed cohort, only data relating to hospital in-patients will be included.

Exclusion criteria:

- studies not written in English
- studies where the sample are non-hospital in-patients (i.e. outpatients, day patients or community-dwelling individuals)

Systematic Review - Screening procedure

1). Import attached RIS file into your reference manager software (preferably EndNote X8.2)

2). Once imported, scan all title and abstracts for eligibility against the inclusion / exclusion criteria above.

3). Move all studies identified as potentially eligible based on title and abstract into a separate group (EndNote), Or folder (RefWorks, Mendeley).

4). Screen full text of identified studies to determine eligibility.

5). Move all eligible studies into separate group / folder.

6). Make note of excluded studies and reasons for their exclusion based on eligibility criteria, in the attached excel file.

5). Compare identified studies.

6). If all reviewers identify the exact same studies, with no discrepancies, this is the end of the initial screening process for the systematic review.

7). If there are differences in the studies identified by different reviewers - discuss until resolution is determined. In the event a unanimous consensus cannot be met by the three reviewers, the majority consensus will be taken, and a note made of this.

For peer review only - http://bmjopen.bmj.com/site/about/guidelines.xhtml

Author	Title	Initial studies included from full text screening (prior to reviewer discussion)		
		Reviewer 1 (PD)	Reviewer 2 (JA)	Reviewer 3 (EA)

For peer review only

Conclusion of discussion	Decision	Response received from author	Outcome

For peer review only

Key

**Unanimous consensus inclusion**	
**Majority consensus inclusion**	
**Minority consensus inclusion / contact author***	*In event where either there is not majority consensus inclusion or exclusion i.e. one reviewer wishes to include and at least one other wishes to seek further information, or two reviewers wish to seek futher information
**Majority consensus exclusion****	** = Reasons for all excluded studies are given in exclusion form
**Unanimous consensus exclusion****	** = Reasons for all excluded studies are given in exclusion form
Unanimous consensus contact author	
? = Contact author***	** = Reasons for all instances of contacting the study author for clarification or futher information to assess eligibility are outlined in contact author form

✓ = Included

x = Excluded

peer review only

JBI Critical Appraisal Checklist for Studies Reporting Prevalence Data

Reviewer: ______________________________ Date: __________________________

Author: _______________________________ Year: __________ Record Number: _______

	Yes	No	Unclear	Not applicable
1. Was the sample representative of the target population?	☐	☐	☐	☐
2. Were study participants recruited in an appropriate way?	☐	☐	☐	☐
3. Was the sample size adequate?	☐	☐	☐	☐
4. Were the study subjects and the setting described in detail?	☐	☐	☐	☐
5. Was the data analysis conducted with sufficient coverage of the identified sample?	☐	☐	☐	☐
6. Were objective, standard criteria used for the measurement of the condition?	☐	☐	☐	☐
7. Was the condition measured reliably?	☐	☐	☐	☐
8. Was there appropriate statistical analysis?	☐	☐	☐	☐
9. Are all important cofounding factors/ subgroups/ differences identified and accounted for?	☐	☐	☐	☐
10. Were subpopulations identified using objective criteria?	☐	☐	☐	☐

Overall appraisal: Include ☐ Exclude ☐ Seek further info ☐

____

____

____

____

For peer review only

Critical appraisal tool guidelines

Answer: Yes, No, Unclear or Not/Applicable.

1. Was the sample representative of the target population?

This question relies upon knowledge of the broader characteristics of the population of interest. If the study is of women with breast cancer, knowledge of at least the characteristics, demographics and medical history is needed. The term "target population" should not be taken to infer every individual from everywhere or with similar disease or exposure characteristics. Instead, give consideration to specific population characteristics in the study, including age range, gender, morbidities, medications, and other potentially influential factors. For example, a sample may not be representative of the target population if a certain group has been used (such as those working for one organisation, or one profession) and the results then inferred to the target population (i.e. working adults).

2. Were study participants recruited in an appropriate way?

Recruitment is the calling or advertising strategy for gaining interest in the study, and is not the same as sampling. Studies may report random sampling from a population, and the methods section should report how sampling was performed. What source of data were study participants recruited from? Was the sampling frame appropriate? For example, census data is a good example of appropriate recruitment as a good census will identify everybody. Was everybody included who should have been included? Were any groups of persons excluded? Was the whole population of interest surveyed? If not, was random sampling from a defined subset of the population employed? Was stratified random sampling with eligibility criteria used to ensure the sample was representative of the population that the researchers were generalizing to?

3. Was the sample size adequate?

An adequate sample size is important to ensure good precision of the final estimate. Ideally we are looking for evidence that the authors conducted a sample size calculation to determine an adequate sample size. This will estimate how many subjects are needed to produce a reliable estimate of the measure(s) of interest. For conditions with a low prevalence, a larger sample size is needed. Also consider sample sizes for subgroup (or characteristics) analyses, and whether these are appropriate. Sometimes, the study will be large enough (as in large national surveys) whereby a sample size calculation is not required. In these cases, sample size can be considered adequate.

When there is no sample size calculation and it is not a large national survey, the reviewers may consider conducting their own sample size analysis using the following formula:[15, 16]

N = Z2P(1-P)

d2

Where:

- N = sample size
- Z = Z statistic for a level of confidence
- P = Expected prevalence or proportion (in proportion of one; if 20%, P = 0.2)
- d = precision (in proportion of one; if 5%, d=0.05)

4. Were the study subjects and setting described in detail?

Certain diseases or conditions vary in prevalence across different geographic regions and populations (e.g. women vs. men, sociodemographic variables between countries). Has the study sample been described in sufficient detail so that other researchers can determine if it is comparable to the population of interest to them?

5. Is the data analysis conducted with sufficient coverage of the identified sample?

A large number of dropouts, refusals or "not founds" amongst selected subjects may diminish a study's validity, as can low response rates for survey studies.

- Did the authors describe the reasons for non-response and compare persons in the study to those not in the study, particularly with regards to their socio-demographic characteristics?

For peer review only - http://bmjopen.bmj.com/site/about/guidelines.xhtml

- Could the not-responders have led to an underestimate of prevalence of the disease or condition under investigation?
- If reasons for non-response appear to be unrelated to the outcome measured and the characteristics of non-responders are comparable to those in the study, the researchers may be able to justify a more modest response rate.
- Did the means of assessment or measurement negatively affect the response rate (measurement should be easily accessible, conveniently timed for participants, acceptable in length and suitable in content).

6. Were objective, standard criteria used for measurement of the condition?

Here we are looking for measurement or classification bias. Many health problems are not easily diagnosed or defined, and some measures may not be capable of including or excluding appropriate levels or stages of the health problem. If the outcomes were assessed based on existing definitions or diagnostic criteria, then the answer to this question is likely to be yes. If the outcomes were assessed using observer reported, or self-reported scales, the risk of over-or under-reporting is increased, and objectivity is compromised. Importantly, determine if the measurement tools used were validated instruments as this has a significant impact on outcome assessment validity.

7. Was the condition measured reliably?

Considerable judgment is required to determine the presence of some health outcomes. Having established the objectivity of the outcome measurement instrument (see item 6 of this scale), it is important to establish how the measurement was conducted. Were those involved in collecting data trained or educated in the use of the instrument/s? If there was more than one data collector, were they similar in terms of level of education, clinical or research experience, or level of responsibility in the piece of research being appraised?
- Has the researcher justified the methods chosen?
- Has the researcher made the methods explicit? (For interview method, how were interviews conducted?)

8. Was there appropriate statistical analysis?

As with any consideration of statistical analysis, consideration should be given to whether there was a more appropriate alternate statistical method that could have been used. The methods section should be detailed enough for reviewers to identify the analytical technique used and how specific variables were measured. Additionally, it is also important to assess the appropriateness of the analytical strategy in terms of the assumptions associated with the approach as differing methods of analysis are based on differing assumptions about the data and how it will respond. Prevalence rates found in studies only provide estimates of the true prevalence of a problem in the larger population. Since some subgroups are very small, 95% confidence intervals are usually given.

9. Are all important confounding factors/ subgroups/differences identified and accounted for?

Incidence and prevalence studies often draw or report findings regarding the differences between groups. It is important that authors of these studies identify all important confounding factors, subgroups and differences and account for these.

10. Were subpopulations identified using objective criteria?

Objective criteria should also be used where possible to identify subgroups (refer to question 6).

If a study scores less than 5/10 (50%) it should be excluded, unless there is ambiguity with relation to the aforementioned criteria, in which case more information should be sought and then the criteria rereviewed.

For peer review only

				Study details								Study Methods		Subject characteristics						
Study	Author	Year of Publication	Study title	Journal of publication	Aim	Setting	Ward / Department / Unit / Hospital / Clinical population type	Study design	Recruitment duration	Age of participants (mean +/-SD)	Age of participants (range)	Sex (proportion male / female participants)	Country / location	Continent	5 year average GDP per capita PPP (current international $) (years proceeding the study) (International	5 year average healthcare expenditure per capita PPP (current international $) (years proceeding the study) (World	Sample size (n)	Diagnosis / Prevalent morbidity (if applicable)	Any other relevant characteristic	Criteria utilised for the operational definition of frailty
Study 2																				
Study 3																				
Study 4																				
Study 5																				
Study 6																				
Study 7																				
Study 8																				
Study 9																				
Study 10																				
Study 11																				
Study 12																				
Study 13																				
Study 14																				
Study 15																				
Study 16																				
Study 17																				
Study 18																				
Study 19																				
Study 20																				
Study 21																				
Study 22																				
Study 23																				

For peer review only

Results

Planned stratified

Number of frail participants	Number of "pre-frail" participants	Number of robust / non-frail	Prevalence of frailty	Prevalence of "pre-frailty"	Prevalence of robust/non frail	Number of Male participants	Number of frail Male participants	Number of pre-frail Male participants	Number of non-frail / robust Male participants	Prevalence of frailty in Male participants	Prevalence of pre-frailty in Male participants	Prevalence of robust / non-frailty Male participants	Number of Female participants	Number of frail Female partcipants	Number of pre-frail Female participants	Number of non-frail / robust Female participants	Prevalence of frailty in Female participants	Prevalence of pre-frailty in Female participants	Prevalence of robust / non-frialty Female participants	Relevant authors comments	Relevant reviewers comments

For peer review only

					5 year average GDP per capita PPP (current international $) (years proceeding the study*) (International Monetary Fund data)																				*5 years prior to commencement of data collection for the study. Each calender year of the study will also be included provided recruitment continues through to > 6 months in the proceeding year.
Author	Year of Publication	Country / location	Recruitment start date	Recruitment end date	Year 5	Year 4	Year 3	Year 2	Year 1	Additional Year 1	Additional year 2	Additional year 3	Additional year 4	Additional year 5	Additional year 6	Additional year 7	Additional year 8	Additional year 9	Additional year 10	Additional year 11	Additional year 12	Years	5 year average GDP per capita PPP (current international $) (years proceeding the study*) (Internation Monetary Fund data)		

For peer review only

					5 year average healthcare expenditure per capita PPP (current international $) (years proceeding the study) (World Health Organisation data)																			
Author	Year of Publicatio n	Country / location	Recruitment start date	Recruitment end date	Year 5	Year 4	Year 3	Year 2	Year 1	Additional Year 1	Additional year 2	Additional year 3	Additional year 4	Additional year 5	Additional year 6	Additional year 7	Additional year 8	Additional year 9	Additional year 10	Additional year 11	Additional year 12	Years	5 year average health care expenditure per capita PPP (current international $) (years proceeding the study*) (World Health Organisation data)	

*5 years prior to commencement of data collection for the study. Each calender year of the study will also be included provided recruitment continues through to > 6 months in the proceeding year.

For peer review only

PRISMA-P (Preferred Reporting Items for Systematic review and Meta-Analysis Protocols) 2015 checklist: recommended items to address in a systematic review protocol*

Section and topic	Item No	Checklist item	Page no.
**ADMINISTRATIVE INFORMATION**			
Title:			
Identification	1a	Identify the report as a protocol of a systematic review	1
Update	1b	If the protocol is for an update of a previous systematic review, identify as such	
Registration	2	If registered, provide the name of the registry (such as PROSPERO) and registration number	2
Authors:			
Contact	3a	Provide name, institutional affiliation, e-mail address of all protocol authors; provide physical mailing address of corresponding author	1
Contributions	3b	Describe contributions of protocol authors and identify the guarantor of the review	1,11
Amendments	4	If the protocol represents an amendment of a previously completed or published protocol, identify as such and list changes; otherwise, state plan for documenting important protocol amendments	
Support:			
Sources	5a	Indicate sources of financial or other support for the review	11
Sponsor	5b	Provide name for the review funder and/or sponsor	11
Role of sponsor or funder	5c	Describe roles of funder(s), sponsor(s), and/or institution(s), if any, in developing the protocol	
**INTRODUCTION**			
Rationale	6	Describe the rationale for the review in the context of what is already known	4
Objectives	7	Provide an explicit statement of the question(s) the review will address with reference to participants, interventions, comparators, and outcomes (PICO)	4,5
**METHODS**			
Eligibility criteria	8	Specify the study characteristics (such as PICO, study design, setting, time frame) and report characteristics (such as years considered, language, publication status) to be used as criteria for eligibility for the review	4,5
Information sources	9	Describe all intended information sources (such as electronic databases, contact with study authors, trial registers or other grey literature sources) with planned dates of coverage	5,6,7
Search strategy	10	Present draft of search strategy to be used for at least one electronic database, including planned limits, such that it could be repeated	Appendix 1
Study records:			

For peer review only

Data management	11a	Describe the mechanism(s) that will be used to manage records and data throughout the review	6,7,8
Selection process	11b	State the process that will be used for selecting studies (such as two independent reviewers) through each phase of the review (that is, screening, eligibility and inclusion in meta-analysis)	6,7,8,9,10
Data collection process	11c	Describe planned method of extracting data from reports (such as piloting forms, done independently, in duplicate), any processes for obtaining and confirming data from investigators	8,9
Data items	12	List and define all variables for which data will be sought (such as PICO items, funding sources), any pre-planned data assumptions and simplifications	8
Outcomes and prioritization	13	List and define all outcomes for which data will be sought, including prioritization of main and additional outcomes, with rationale	9,10
Risk of bias in individual studies	14	Describe anticipated methods for assessing risk of bias of individual studies, including whether this will be done at the outcome or study level, or both; state how this information will be used in data synthesis	7,8
Data synthesis	15a	Describe criteria under which study data will be quantitatively synthesised	7,8
	15b	If data are appropriate for quantitative synthesis, describe planned summary measures, methods of handling data and methods of combining data from studies, including any planned exploration of consistency (such as I^2, Kendall's τ)	9,10
	15c	Describe any proposed additional analyses (such as sensitivity or subgroup analyses, meta-regression)	9,10
	15d	If quantitative synthesis is not appropriate, describe the type of summary planned	8
Meta-bias(es)	16	Specify any planned assessment of meta-bias(es) (such as publication bias across studies, selective reporting within studies)	10
Confidence in cumulative evidence	17	Describe how the strength of the body of evidence will be assessed (such as GRADE)	

*** It is strongly recommended that this checklist be read in conjunction with the PRISMA-P Explanation and Elaboration (cite when available) for important clarification on the items. Amendments to a review protocol should be tracked and dated. The copyright for PRISMA-P (including checklist) is held by the PRISMA-P Group and is distributed under a Creative Commons Attribution Licence 4.0.**

From: Shamseer L, Moher D, Clarke M, Ghersi D, Liberati A, Petticrew M, Shekelle P, Stewart L, PRISMA-P Group. Preferred reporting items for systematic review and meta-analysis protocols (PRISMA-P) 2015: elaboration and explanation. BMJ. 2015 Jan 2;349(jan02 1):g7647.

For peer review only

BMJ Open

Frailty Levels in Geriatric Hospital paTients (FLIGHT) - The prevalence of frailty amongst geriatric populations within hospital ward settings: A systematic review protocol

Journal:	*BMJ Open*
Manuscript ID	bmjopen-2019-030147.R2
Article Type:	Protocol
Date Submitted by the Author:	01-Jul-2019

Complete List of Authors:	Doody, Paul; University of Birmingham, School of Sport, Exercise and Rehabilitation Sciences<br>Aunger, Justin; University of Birmingham, School of Sport, Exercise and Rehabilitation Sciences<br>Asamane, Evans; University of Birmingham, School of Sport, Exercise and Rehabilitation Sciences<br>Greig, Carolyn; School of Sport, Exercise and Rehabilitation Sciences, University of Birmingham, Edgbaston, Birmingham, B15 2TT, UK; MRC-Arthritis Research UK Centre for Musculoskeletal Ageing Research<br>Lord, Janet; University of Birmingham, NIHR Surgical Reconstruction & Microbiology Research Centre<br>Whittaker, Anna; University of Birmingham, School of Sport, Exercise and Rehabilitation Sciences; University of Stirling

**Primary Subject Heading**:	Geriatric medicine
Secondary Subject Heading:	Geriatric medicine, Health economics
Keywords:	Frail, Geriatric, inpatient, older adult, prevalence, systematic review

SCHOLARONE™
Manuscripts

Protocol_Manuscript_V3.0_01/07/2019

Frailty Levels in Geriatric Hospital paTients (FLIGHT) - The prevalence of frailty amongst geriatric populations within hospital ward settings: A systematic review protocol

Paul Doody[1], Justin A. Aunger[1], Evans A. Asamane[1], Carolyn A. Greig[1,2,3], Janet M. Lord[2], Anna C. Whittaker[1,4]

[1] School of Sport, Exercise and Rehabilitation Sciences, University of Birmingham, United Kingdom

[2] Institute of Inflammation and Ageing MRC-ARUK Centre for Musculoskeletal Ageing Research, University of Birmingham, United Kingdom

[3] NIHR Birmingham Biomedical Research Centre, University Hospitals Birmingham NHS Foundation Trust and University of Birmingham, United Kingdom

[4] Faculty of Health Sciences and Sport, University of Stirling, United Kingdom

Corresponding author: Paul Doody (p.d.doody@bham.ac.uk, +44 (0) 121 414 4125), School of Sport Exercise and Rehabilitation Sciences (G35), University of Birmingham, United Kingdom

For peer review only

Abstract

Introduction: Frailty is a common and clinically significant condition in geriatric populations, associated with adverse health outcomes such as hospitalisation, disability, and mortality. Although there are systematic reviews/meta-analyses assessing the prevalence of frailty in community-dwelling older adults, nursing home residents, and cancer and general surgery patients, there are none assessing the overall prevalence of frailty in geriatric hospital inpatients.

Methods and analysis: This review will systematically search and analyse the prevalence of frailty within geriatric hospital inpatients within the literature. A search will be employed on the platforms of Ovid, Web of Science, and databases of CINAHL Plus, SCOPUS, and the Cochrane Library. Any observational or experimental study design which utilises a validated operational definition of frailty, reports the prevalence of frailty, has a minimum age ≥ 65 years, attempts to assess the whole ward/clinical population, and occurs in hospital inpatients, will be included. Title and abstract and full-text screenings will be conducted by three reviewers. Methodological quality of eligible studies will be assessed utilising the Joanna Briggs Institute critical appraisal tool. Data extraction will be performed by two reviewers. If sufficient data are available, a meta-analysis synthesising pooled estimates of the prevalence of frailty and pre-frailty, as well as the prevalence of frailty stratified by age, sex, operational frailty definition, prevalent morbidities, ward type, and location, among older hospitalised in-patients will be conducted. Clinical heterogeneity will be assessed by two reviewers. Statistical heterogeneity will be assessed through a Cochran Q test, and an I^2 test performed to assess its magnitude.

Ethics and dissemination: Ethical approval was not required as primary data will not be collected. Findings will be disseminated through publication in peer reviewed open access scientific journals, public engagement events, conference presentations, and social media.

Trial Registration number: This study has been registered on PROSPERO (registration number 79202).

Strengths and limitations of this study:

For peer review only - http://bmjopen.bmj.com/site/about/guidelines.xhtml

- First review to systematically or exclusively assess the overall prevalence of frailty in geriatric hospital inpatients
- Will seek to provide stratified analysis of the prevalence of frailty based on age, sex, operational frailty definition, prevalent morbidities, ward type and location
- Three independent reviewers during screening phase; ensuring high internal reliability and consistency of included studies
- Will include only studies for which the full text is available in English, therefore will likely be relatively over-representative of Western nations (Europe, Australasia, and the Americas); although this is true of scientific publications in general.
- **Keywords**: department; frail; geriatric; hospital; inpatient; meta-analysis; older adult; prevalence; systematic review; ward.

For peer review only

Introduction

Frailty is a common and clinically significant condition within geriatric populations (1), predominantly due to its association with adverse health outcomes such as hospitalization, disability and mortality (1-6). Although there are systematic reviews and meta-analysis assessing the prevalence of frailty amongst community-dwelling older adults (7-10), nursing home residents (11), and cancer (12) and general surgery patients (13), presently there are no systematic reviews or meta-analysis which assess the overall prevalence of frailty among geriatric hospital inpatients. This constitutes an important gap in the literature which needs to be addressed and has important consequences. Such consequences include the tailoring of services within this setting to the needs of service users, for example, the potential implementation of exercise rehabilitation treatments within this setting for this cohort; with physical activity and exercise being proposed as potentially offering the best form of treatment for frail older adults (14), and shown to be capable of reducing, and even reversing frailty within older adults (15,16). Through providing a highly detailed analysis of the prevalence of frailty amongst older population within this setting, this review has the potential to aid in the facilitation of improvements in the planning and orientation of organisational structures and resources, to meet the needs of this population, and enhance the care of frail older adults in inpatient hospital settings.

Methods and Design

Review Aim:

The aim of this review is to systematically search and analyse the prevalence of frailty amongst geriatric populations (aged ≥ 65 years) within inpatient hospital settings within the literature. If a meta-analysis proves possible, the aim of this study is also to synthesise pooled estimates of the prevalence of frailty and pre-frailty, as well as the prevalence of frailty stratified by age, sex, operational frailty definition, prevalent morbidities, ward type and location (country and continent), among hospital in-patients.

For peer review only - http://bmjopen.bmj.com/site/about/guidelines.xhtml

Review Objectives:

1) To identify and compare studies reporting the prevalence of frailty within hospital ward settings.
2) To combine the extracted data to calculate the pooled overall prevalence of frailty in hospitalised geriatric in-patients.
3) To perform stratified analysis of the prevalence of frailty based on age, sex, operational frailty definition, prevalent morbidity and ward type in order to assess the relationship between frailty and these factors.

Eligibility criteria:

Inclusion criteria: all studies must have a minimum age of ≥ 65 years, use a clearly defined and validated operational definition for the classification of frailty (i.e. has been specifically validated for the assessment of frailty, either through comparison with existing validated tools, or its predictive value regarding negative health outcomes aligned with frailty), either assess (or attempt to assess) the whole ward, department, unit, hospital or specific clinical population, or employ some form of randomised selection of participants, occur within a hospital setting, in, or including, hospital in-patients (operationally defined as any patient admitted to hospital who remains overnight, or were initially expected to remain overnight), report the prevalence of frailty or provide sufficient data to allow the calculation of the prevalence of frailty. If a study examines a mixed cohort, only data relating to hospital in-patients will be included in the review.

Exclusion criteria: all studies not written in English, studies where the sample are not hospital inpatients (i.e. outpatients, day patients or community-dwelling individuals).

Information sources:

Searches will be conducted on the platforms of Ovid (incorporating the databases of Journals @Ovid full text, EMBASE, CAB abstracts, Ovid MEDLINE ® In process and other non-indexed citations, Ovid MEDLINE ®, and PyschINFO) and Web of Science (incorporating the databases of Science Citation Index Expanded (SCI-Expanded), Conference Proceedings Citation Index – Science (CRI-S), and Emerging

For peer review only - http://bmjopen.bmj.com/site/about/guidelines.xhtml

Sources Citation Index (ESCI)), and the databases of CINAHL Plus, SCOPUS, and the Cochrane Library databases (the Cochrane Database of Systematic Reviews (CDSR), the Cochrane Central Register of Controlled Trials (CENTRAL), the Cochrane Methodology Register (CMR), the Database of Abstracts of Reviews of Effect (DARE), Health Technology Assessment database (HTA) and the NHS Economic Evaluation Database (EED)).

Types of studies:

Any form of observational or experimental study design which assesses the prevalence of frailty and meets the above eligibility criteria. For longitudinal observational studies, and experimental studies, frailty scores and additional data will be extracted from baseline data, provided baseline data meets the above eligibility criteria.

Search Strategy:

The search strategy will be conducted on the two platforms of Ovid and Web of Science, as well as the databases of SCOPUS, CINAHL Plus, and the Cochrane Library databases (Appendix 1). These searches will encompass all available literature published prior to 21/11/2018.

Screening:

Prior to the commencement of title and abstract screening by the three independent reviewers, duplicates will be removed utilising EndNote X8.2. The reduced list of studies will be manually screened for the removal of any remaining duplicates. All reviewers will be provided with an instructional screening form (Appendix 2), and a .ris file containing all studies captured within database searches. The screening form will list the eligibility criteria and instructions on setting up the .ris file for screening within a reference manager.

For peer review only - http://bmjopen.bmj.com/site/about/guidelines.xhtml

The title and abstract of all studies will then be independently screened by the three reviewers, with each reviewer placing potentially eligible studies into a separate folder. Upon completion, potentially eligible studies from all three reviewers will be placed into a "master folder" and the results collated. Duplicates will be removed, leaving the final combined list of studies for the full text screening phase. All reviewers will then independently screen the full text of remaining studies utilising the screening form and maintain separate files for included and excluded studies (including reasons), as well as for studies for which the reviewer feels the need to contact the authors for clarification or additional information.

Upon completion, a full text screening master file (Appendix 3) will be formulated by the lead reviewer displaying each reviewer's full text screening decision for each study. All three reviewers will then meet to discuss the decisions of each study and endeavour to come to an agreement on studies for which there is not initial unanimous consensus. During this process a full list of included and excluded studies (with reasons), and studies for which reviewers agree to contact authors for additional information or clarification will be formed by the lead reviewer. The lead reviewer will then contact study authors and, upon receipt of clarification or additional information, will meet with reviewers to discuss the inclusion/exclusion of the study.

Manual screening will also be employed by reviewers and include the reference lists of all included studies, as well as excluded but potentially relevant studies or systematic reviews captured within the screening. As part of the grey literature search of this review, in process publications will also be searched and conference abstracts will be followed up with authors to ascertain if a full text relating to the data is available. Studies of the same cohort will be included only once, using the study which provides the most information about the cohort relevant to this review.

Assessment of methodological quality:

The quality of eligible studies from full text screening will be assessed by two reviewers independently using the Joanna Briggs Institute (JBI) critical appraisal tool for studies reporting prevalence data (17)

For peer review only - http://bmjopen.bmj.com/site/about/guidelines.xhtml

(Appendix 4). In the event of any discrepancies between the two reviewers, a consensus will be attempted to be reached by discussion. In the event a full consensus cannot be reached between the two reviewers after an exhaustive discussion, the opinion of a third reviewer will be obtained, and the proceeding majority consensus will be taken.

Data extraction:

Data extraction will be performed by two reviewers independently. In the event of any discrepancies between the two reviewers, a consensus will be attempted to be reached by discussion. In the event that a full consensus cannot be reached between the two reviewers after an exhaustive discussion, the opinion of a third reviewer will be obtained, and the proceeding majority consensus will be taken.

The following data, where available, will be extracted from all eligible studies (see Appendix 5 for template). If any data are not immediately available, the authors of the studies in question will be contacted in an attempt to retrieve all applicable data:

Study details: authors, year of publication, study title, journal of publication, aim. Study methods: setting, ward/department/unit/hospital type/clinical population, study design, recruitment duration, subject characteristics (age of participants (mean and standard deviation, range)), sex (proportion of male / female participants), country / continent, sample size, diagnosis / prevalent morbidity (if applicable), any other relevant characteristics), criteria utilised for the operational definition of frailty. Results: Number of frail participants, number of "pre-frail" participants, number of robust / non-frail participants, prevalence of frailty, prevalence of pre-frailty, prevalence of robustness / non-frailty, number of male participants, number of frail male participants, number of pre-frail male participants, number of non-frail / robust male participants, prevalence of frailty in male participants, prevalence of pre-frailty in male participants, prevalence of non-frailty / robustness in male participants, number of female participants, number of frail female participants, number of pre-frail female participants, number of non-frail / robust female participants, prevalence of frailty in female participants, prevalence of pre-frailty in female participants, prevalence of non-frailty / robustness in female participants, and finally authors comments and reviewers' comments.

For peer review only - http://bmjopen.bmj.com/site/about/guidelines.xhtml

External to the studies, data will also be extracted with regard to the 5 year average Gross Domestic Product (GDP) per capita Purchasing Power Parity (PPP) (current international $) of the country in which each study takes place, incorporating the five years directly preceding the commencement of recruitment to the study (18). External data will also be extracted with regard to the 5 year average health care expenditure per capita PPP (current international $) of the country in which each study takes place, incorporating the five years directly preceding the commencement of recruitment to the study (19). Each calendar year of the study will also be included provided recruitment continues through to > 6 months in the preceding year.

Data synthesis:

Quantitative synthesis (Meta-Analysis): If a sufficient quantity of identified studies are comparable, a meta-analysis, pooling the aggregated data from each study, will be performed. Clinical heterogeneity will be assessed by two reviewers based on their judgement of the available data and any disagreements will be discussed thoroughly with the aim of reaching a unanimous consensus. If a unanimous consensus cannot be reached, the opinion of a third reviewer will be sought, and the proceeding majority consensus will be taken. Statistical heterogeneity will be assessed through the utilisation of a Cochran Q test and considered present at $p < .05$. An I^2 test will be performed in order to assess the magnitude of this heterogeneity, with I^2 values of 25%, 50% and 75% being considered low, moderate and high respectively. If the Cochrane Q statistic test detected statistically significant heterogeneity, combined with the researcher's assessment, a randomised-effects model will be utilised. Given the nature of this review and in particular its overall aim, combined with the eligible studies identified in preliminary searches, it is likely the initial quantitative synthesis will utilise a random-effects model.

Stratified analysis will also be conducted according to age (65 – 74 years, 75 – 84 years and 85+ years), sex, operational frailty definition, ward type, prevalent morbidity and location (country and continent) where possible. These variables have been specifically chosen for stratified analysis predominantly due to an enhanced knowledge of these areas being of practical utility to researchers and clinicians; stemming from empirical evidence persistently showing alterations in these factors to impact upon the prevalence of frailty

For peer review only - http://bmjopen.bmj.com/site/about/guidelines.xhtml

(2,4,20-22). As such stratified analysis pertaining to these variables will facilitate this review to provide a more in-depth and thorough insight into the prevalence of frailty amongst geriatric hospital inpatients.

Clinical heterogeneity for stratified analysis will be assessed by two reviewers based on their judgement of the available data. Any disagreements will be discussed thoroughly with the aim of reaching a unanimous consensus. If a unanimous consensus cannot be reached, the opinion of a third reviewer will be sought. Statistical heterogeneity for sub-analysis will similarly be assessed through the utilisation of a Cochran Q test and considered present at $p < .05$. An I^2 test will be performed in order to assess the magnitude of this heterogeneity, with I^2 values of 25%, 50% and 75% being considered low, moderate and high respectively.

Similarly, it is likely a random-effects model will be utilised to synthesise pooled estimates of the prevalence of frailty stratified by these criteria (although there is more of a likelihood that a fixed effects model could potentially be utilised within these analyses, in comparison to the initial analysis, given the nature of stratified analysis).

Correlation analysis will also be employed to examine the relationship between the prevalence of frailty of geriatric inpatients and economic prosperity (GDP per capita PPP) (current international $), and health care expenditure (per capita PPP) (current international $). Additionally, multi-linear regression analysis will examine the predictive value between economic prosperity and health care expenditure, and the prevalence of frailty of geriatric inpatients.

Qualitative synthesis: if a meta-analysis is not possible based on the nature of the studies and the data available, a more thorough systematic narrative analysis will be conducted, with findings presented in both textual and tabular formats.

Patient and Public involvement

All authors are strong proponents of patient and public involvement and engagement with research and believe the finding of this review will be important to aid the facilitation of improvements in the planning

For peer review only - http://bmjopen.bmj.com/site/about/guidelines.xhtml

and orientation of organisation structures and resources within this setting to meet the needs of service users; specifically relating to the enhanced care of older adults in inpatient hospital settings. However, given the nature of this study (systematic review), it was not possible to involve the public. However, the findings will be disseminated to our patient and public involvement groups.

Ethics and Dissemination

Formal ethical approval was not required for this review as primary data will not be collected. The findings of this study will be disseminated through publication in the form of scientific papers in peer reviewed open access scientific journals, public engagement events within the United Kingdom and Europe, online via social media (Twitter, Instagram) and the PANINI project website (23,24), and presentation at conferences within the UK and internationally. This review is scheduled for completion during the second half of 2019.

Funding

This review has been funded by the European Commission's Horizon 2020 research and innovation programme under the Marie Sklodowska-Curie grant agreement (675003); of which PD, EA and JA are Marie Sklodowska-Curie Doctoral Research Fellows, AW, JL and CG doctoral supervisors, and AW the grants Principal Investigator.

Author contributions

PD is guarantor and lead reviewer. PD designed the systematic review protocol, conducted the literature searches and prepared this manuscript for publication, with supervision, input and feedback from AW, CG and JL. EA and JA are independent reviewers for title and abstract and full text screenings. JA will also act as independent data extractor for included studies. All authors have read and approved the final manuscript.

For peer review only - http://bmjopen.bmj.com/site/about/guidelines.xhtml

Conflicts of interest:

The authors of this review report no known conflicts of interest.

Acknowledgements:

The authors of this review would like to thank and acknowledge Ms. Lynne Harris (Subject advisor of the Main Library at the University of Birmingham, United Kingdom) for her assistance during the formulation of the search strategy utilised within this systematic review.

Word count (excluding title page, abstract, author contributions, acknowledgements and references): 2507

For peer review only

References:

(1) Rodriguez-Mañas L, Fried LP. Frailty in the clinical scenario. The Lancet 2015 /02/14;385(9968):e9.

(2) Fried LP, Tangen CM, Walston J, Newman AB, Hirsch C, Gottdiener J, et al. Frailty in older adults evidence for a phenotype. The Journals of Gerontology Series A: Biological Sciences and Medical Sciences 2001;56(3):M157.

(3) Gill TM, Gahbauer EA, Allore HG, Han L. Transitions between frailty states among community-living older persons. Arch Intern Med 2006;166(4):418-423.

(4) Sternberg SA, Schwartz AW, Karunananthan S, Bergman H, Mark Clarfield A. The identification of frailty: a systematic literature review. J Am Geriatr Soc 2011;59(11):2129-2138.

(5) Clegg A, Young J, Iliffe S, Rikkert MO, Rockwood K. Frailty in elderly people. The Lancet 2013 March 8,;381(9868):752-762.

(6) Sourial N, Bergman H, Karunananthan S, Wolfson C, Payette H, Gutierrez-Robledo LM, et al. Implementing Frailty Into Clinical Practice: A Cautionary Tale. J Gerontol A Biol Sci Med Sci 2013 -05-02:glt053.

(7) Collard RM, Boter H, Schoevers RA, Oude Voshaar RC. Prevalence of frailty in community-dwelling older persons: a systematic review. J Am Geriatr Soc 2012 Aug;60(8):1487-1492.

(8) Siriwardhana DD, Hardoon S, Rait G, Weerasinghe MC, Walters KR. Prevalence of frailty and prefrailty among community-dwelling older adults in low-income and middle-income countries: a systematic review and meta-analysis. BMJ open 2018;8(3):e018195.

(9) Kojima G, Iliffe S, Taniguchi Y, Shimada H, Rakugi H, Walters K. Prevalence of frailty in Japan: A systematic review and meta-analysis. Journal of epidemiology 2017;27(8):347-353.

(10) Verlaan S, Ligthart-Melis GC, Wijers SL, Cederholm T, Maier AB, de van der Schueren, Marian AE. High prevalence of physical frailty among community-dwelling malnourished older adults–a systematic review and meta-analysis. Journal of the American Medical Directors Association 2017;18(5):374-382.

(11) Kojima G. Prevalence of frailty in nursing homes: A systematic review and meta-analysis. Journal of the American Medical Directors Association 2015;16(11):940-945.

(12) Handforth C, Clegg A, Young C, Simpkins S, Seymour MT, Selby PJ, et al. The prevalence and outcomes of frailty in older cancer patients: a systematic review. Annals of Oncology 2015 June;26(6):1091-1101.

(13) Hewitt J, Long S, Carter B, Bach S, McCarthy K, Clegg A. The prevalence of frailty and its association with clinical outcomes in general surgery: a systematic review and meta-analysis. Age Ageing 2018;47(6):793-800.

(14) Theou O, Stathokostas L, Roland KP, Jakobi JM, Patterson C, Vandervoort AA, et al. The effectiveness of exercise interventions for the management of frailty: a systematic review. Journal of aging research 2011;2011.

(15) Fiatarone MA, O'Neill EF, Ryan ND, Clements KM, Solares GR, Nelson ME, et al. Exercise training and nutritional supplementation for physical frailty in very elderly people. N Engl J Med 1994;330(25):1769-1775.

(16) Tarazona-Santabalbina FJ, Gmez-Cabrera MC, Prez-Ros P, Martnez-Arnau FM, Cabo H, Tsaparas K, et al. A multicomponent exercise intervention that reverses frailty and improves cognition, emotion, and social networking in the community-dwelling frail elderly: a randomized clinical trial. Journal of the American Medical Directors Association 2016;17(5):426-433.

For peer review only - http://bmjopen.bmj.com/site/about/guidelines.xhtml

(17) Munn Z, Moola S, Riitano D, Lisy K. The development of a critical appraisal tool for use in systematic reviews addressing questions of prevalence. International journal of health policy and management 2014;3(3):123.

(18) International Monetary Fund (. World Economic Outlook (April 2019). 2019; Available at: https://www.imf.org/external/datamapper/PPPPC@WEO/ADVEC/WEOWORLD. Accessed 02/05/, 2019.

(19) World Health Organisation (. Global Health Expenditure Database (GHED). 2019; Available at: http://apps.who.int/nha/database/Home/Index/en. Accessed 02/05/, 2019.

(20) Purser JL, Kuchibhatla MN, Fillenbaum GG, Harding T, Peterson ED, Alexander KP. Identifying frailty in hospitalized older adults with significant coronary artery disease. J Am Geriatr Soc 2006;54(11):1674-1681.

(21) Santos-Eggimann B, Cuénoud P, Spagnoli J, Junod J. Prevalence of frailty in middle-aged and older community-dwelling Europeans living in 10 countries. The Journals of Gerontology: Series A 2009;64(6):675-681.

(22) Andela RM, Dijkstra A, Slaets JPJ, Sanderman R. Prevalence of frailty on clinical wards: Description and implications; 20158543. Int J Nurs Pract 2010;16(1):14-19.

(23) Whittaker AC, Delledonne M, Finni T, Garagnani P, Greig C, Kallen V, et al. Physical Activity and Nutrition INfluences In ageing (PANINI): consortium mission statement. Aging clinical and experimental research 2018:1-8.

(24) University of Birmingham. Physical Activity and Nutritional INfluences In ageing (PANINI) Available at: http://www.birmingham.ac.uk/generic/panini/index.aspx. Accessed 6th February, 2019.

For peer review only

Search Strategy:

Ovid Search Strategy

1. Frail$.ti.ab.
2. Prevalence.ti,ab.
3. Percent$.ti,ab.
4. “were frail”.ti,ab.
5. “considered frail”.ti,ab.
6. Hospital$.ti,ab.
7. Ward.ti,ab.
8. Department.ti,ab.
9. Surg*.ti,ab.
10. Unit.ti,ab.
11. Geriatr*.tx.
12. “older adult*”.tx.
13. Elder$.tx.
14. Retire*.tx.
15. Old$.tx.
16. Patient$.tx.
17. “community-dwelling”.ti,ab.
18. 2 OR 3 OR 4 OR 5
19. 6 OR 7 OR 8 OR 9 OR 10
20. 11 OR 12 OR 13 OR 14 OR 15 OR 16
21. 1 AND 18 AND 19 AND 20
22. 21 NOT 17

Scopus Search Strategy

((((TITLE-ABS-KEY(frail*)) AND (TITLE-ABS-KEY(Prevalence)) OR (TITLE-ABS-KEY(Percent*)) OR (TITLE-ABS-KEY ("were frail")) OR (TITLE-ABS-KEY ("considered frail"))) AND (((TITLE-ABS-KEY(Hospital*)) OR (TITLE-ABS-KEY(Ward)) OR (TITLE-ABS-KEY(Department)) OR (TITLE-ABS-KEY(surg*)) OR (TITLE-ABS-KEY(unit))))) AND ((ALL(Geriatr*)) OR (ALL("older adult*")) OR (ALL(Elder*)) OR (ALL(retire*)) OR (ALL(old)) OR (ALL(older)) OR (ALL(Patient*)))) AND NOT (TITLE-ABS-KEY("community-dwelling"))

Web of Science Search Strategy

1. TS = Frail*
2. TS = Prevalence
3. TS = Percent*
4. TS = “were frail”
5. TS = “considered frail”
6. TS = Hospital*
7. TS = Ward
8. TS = Department
9. TS = Surg*
10. TS = Unit
11. TS = Geriatr*
12. TS = “older adult”
13. TS = Elder*
14. TS = Retir*
15. TS = Old*
16. TS = Patient*
17. TS = “community-dwelling”
18. #2 OR #3 OR #4 OR #5

For peer review only

19. #6 OR #7 OR #8 OR #9 OR #10
20. #11 OR #12 OR #13 OR #14 OR #15 OR #16
21. #1 AND #18 AND #19 AND #20
22. #21 NOT #17

CINAHL PLUS Search Strategy

1. AB frail*
2. AB prevalence OR AN Percent* OR AB "were frail" OR AB "considered frail"
3. AB Hospital* OR AB Ward OR AB Department OR AB Surg* OR AB Unit
4. AB Geriatr* OR AB "older adult" OR AB Elder* OR AB Retir* OR AB OLD* OR AB Patient*
5. S1 AND S2 AND S3 AND S4

Cochrane Library Search Strategy

1. frail*:ti,ab,kw (Word variations have been searched)
2. prevalence:ti,ab,kw or percent*:ti,ab,kw or "were frail":ti,ab,kw or "considered frail":ti.ab.kw (Word variations have been searched)
3. hospital*:ti,ab,kw or ward:ti,ab,kw or department:ti,ab,kw or surg*:ti,ab,kw or unit:ti,ab,kw (Word variations have been searched)
4. Geriatr*:ti,ab,kw or "older adult":ti,ab,kw or Elder*:ti,ab,kw or Retir*:ti,ab,kw or Old*:ti,ab,kw (Word variations have been searched)
5. Patient*:ti,ab,kw (Word variations have been searched)
6. #4 OR #5
7. #1 AND #2 AND #3 AND #6

For peer review only

Frailty Levels In Geriatric Hospital in-paTients (FLIGHT)
Systematic Review Search Strategy Screening form
PANINI (Physical Activity and Nutritional INfluences in Ageing) project
University of Birmingham
2016 - 2019

UNIVERSITY OF BIRMINGHAM

"The prevalence of frailty amongst geriatric populations within hospital ward settings: A systematic review"

Inclusion criteria - All studies must:

- have a minimum age of ≥ 65 years
- use a clearly defined and validated operational definition for the classification of frailty
- either assess (or attempt to assess) the whole ward, department, unit, hospital, or clinical population, or employ some form of randomised selection of participants
- occur within a hospital setting, in, or including hospital in-patients*
- report the prevalence of frailty or provide sufficient data to allow the calculation of the prevalence of frailty.

* If a study examines a mixed cohort, only data relating to hospital in-patients will be included.

Exclusion criteria:

- studies not written in English
- studies where the sample are non-hospital in-patients (i.e. outpatients, day patients or community-dwelling individuals)

Systematic Review - Screening procedure

1). Import attached RIS file into your reference manager software (preferably EndNote X8.2)

2). Once imported, scan all title and abstracts for eligibility against the inclusion / exclusion criteria above.

3). Move all studies identified as potentially eligible based on title and abstract into a separate group (EndNote), Or folder (RefWorks, Mendeley).

4). Screen full text of identified studies to determine eligibility.

5). Move all eligible studies into separate group / folder.

6). Make note of excluded studies and reasons for their exclusion based on eligibility criteria, in the attached excel file.

5). Compare identified studies.

6). If all reviewers identify the exact same studies, with no discrepancies, this is the end of the initial screening process for the systematic review.

7). If there are differences in the studies identified by different reviewers - discuss until resolution is determined. In the event a unanimous consensus cannot be met by the three reviewers, the majority consensus will be taken, and a note made of this.

For peer review only - http://bmjopen.bmj.com/site/about/guidelines.xhtml

Author	Title	Initial studies included from full text screening (prior to reviewer discussion)		
		Reviewer 1 (PD)	Reviewer 2 (JA)	Reviewer 3 (EA)

For peer review only

Conclusion of discussion	Decision	Response received from author	Outcome

For peer review only

Key

Unanimous consensus inclusion

Majority consensus inclusion

Minority consensus inclusion / contact author*

*In event where either there is not majority consensus inclusion or exclusion i.e. one reviewer wishes to include and at least one other wishes to seek further information, or two reviewers wish to seek futher information

Majority consensus exclusion**

** = Reasons for all excluded studies are given in exclusion form

Unanimous consensus exclusion**

** = Reasons for all excluded studies are given in exclusion form

Unanimous consensus contact author

? = Contact author***

** = Reasons for all instances of contacting the study author for clarification or futher information to assess eligibility are outlined in contact author form

✓ = Included

x = Excluded

For peer review only

JBI Critical Appraisal Checklist for Studies Reporting Prevalence Data

Reviewer: ______________________ Date: ______________________

Author: ______________________ Year: __________ Record Number: __________

	Yes	No	Unclear	Not applicable
1. Was the sample representative of the target population?	☐	☐	☐	☐
2. Were study participants recruited in an appropriate way?	☐	☐	☐	☐
3. Was the sample size adequate?	☐	☐	☐	☐
4. Were the study subjects and the setting described in detail?	☐	☐	☐	☐
5. Was the data analysis conducted with sufficient coverage of the identified sample?	☐	☐	☐	☐
6. Were objective, standard criteria used for the measurement of the condition?	☐	☐	☐	☐
7. Was the condition measured reliably?	☐	☐	☐	☐
8. Was there appropriate statistical analysis?	☐	☐	☐	☐
9. Are all important cofounding factors/ subgroups/ differences identified and accounted for?	☐	☐	☐	☐
10. Were subpopulations identified using objective criteria?	☐	☐	☐	☐

Overall appraisal: Include ☐ Exclude ☐ Seek further info ☐

____

____

____

____

For peer review only

Critical appraisal tool guidelines

Answer: Yes, No, Unclear or Not/Applicable.

1. Was the sample representative of the target population?

This question relies upon knowledge of the broader characteristics of the population of interest. If the study is of women with breast cancer, knowledge of at least the characteristics, demographics and medical history is needed. The term "target population" should not be taken to infer every individual from everywhere or with similar disease or exposure characteristics. Instead, give consideration to specific population characteristics in the study, including age range, gender, morbidities, medications, and other potentially influential factors. For example, a sample may not be representative of the target population if a certain group has been used (such as those working for one organisation, or one profession) and the results then inferred to the target population (i.e. working adults).

2. Were study participants recruited in an appropriate way?

Recruitment is the calling or advertising strategy for gaining interest in the study, and is not the same as sampling. Studies may report random sampling from a population, and the methods section should report how sampling was performed. What source of data were study participants recruited from? Was the sampling frame appropriate? For example, census data is a good example of appropriate recruitment as a good census will identify everybody. Was everybody included who should have been included? Were any groups of persons excluded? Was the whole population of interest surveyed? If not, was random sampling from a defined subset of the population employed? Was stratified random sampling with eligibility criteria used to ensure the sample was representative of the population that the researchers were generalizing to?

3. Was the sample size adequate?

An adequate sample size is important to ensure good precision of the final estimate. Ideally we are looking for evidence that the authors conducted a sample size calculation to determine an adequate sample size. This will estimate how many subjects are needed to produce a reliable estimate of the measure(s) of interest. For conditions with a low prevalence, a larger sample size is needed. Also consider sample sizes for subgroup (or characteristics) analyses, and whether these are appropriate. Sometimes, the study will be large enough (as in large national surveys) whereby a sample size calculation is not required. In these cases, sample size can be considered adequate.

When there is no sample size calculation and it is not a large national survey, the reviewers may consider conducting their own sample size analysis using the following formula:[15, 16]

N = Z2P(1-P)

d2

Where:

- N = sample size
- Z = Z statistic for a level of confidence
- P = Expected prevalence or proportion (in proportion of one; if 20%, P = 0.2)
- d = precision (in proportion of one; if 5%, d=0.05)

4. Were the study subjects and setting described in detail?

Certain diseases or conditions vary in prevalence across different geographic regions and populations (e.g. women vs. men, sociodemographic variables between countries). Has the study sample been described in sufficient detail so that other researchers can determine if it is comparable to the population of interest to them?

5. Is the data analysis conducted with sufficient coverage of the identified sample?

A large number of dropouts, refusals or "not founds" amongst selected subjects may diminish a study's validity, as can low response rates for survey studies.

- Did the authors describe the reasons for non-response and compare persons in the study to those not in the study, particularly with regards to their socio-demographic characteristics?

For peer review only

- Could the not-responders have led to an underestimate of prevalence of the disease or condition under investigation?
- If reasons for non-response appear to be unrelated to the outcome measured and the characteristics of non-responders are comparable to those in the study, the researchers may be able to justify a more modest response rate.
- Did the means of assessment or measurement negatively affect the response rate (measurement should be easily accessible, conveniently timed for participants, acceptable in length and suitable in content).

6. Were objective, standard criteria used for measurement of the condition?

Here we are looking for measurement or classification bias. Many health problems are not easily diagnosed or defined, and some measures may not be capable of including or excluding appropriate levels or stages of the health problem. If the outcomes were assessed based on existing definitions or diagnostic criteria, then the answer to this question is likely to be yes. If the outcomes were assessed using observer reported, or self-reported scales, the risk of over-or under-reporting is increased, and objectivity is compromised. Importantly, determine if the measurement tools used were validated instruments as this has a significant impact on outcome assessment validity.

7. Was the condition measured reliably?

Considerable judgment is required to determine the presence of some health outcomes. Having established the objectivity of the outcome measurement instrument (see item 6 of this scale), it is important to establish how the measurement was conducted. Were those involved in collecting data trained or educated in the use of the instrument/s? If there was more than one data collector, were they similar in terms of level of education, clinical or research experience, or level of responsibility in the piece of research being appraised?
- Has the researcher justified the methods chosen?
- Has the researcher made the methods explicit? (For interview method, how were interviews conducted?)

8. Was there appropriate statistical analysis?

As with any consideration of statistical analysis, consideration should be given to whether there was a more appropriate alternate statistical method that could have been used. The methods section should be detailed enough for reviewers to identify the analytical technique used and how specific variables were measured. Additionally, it is also important to assess the appropriateness of the analytical strategy in terms of the assumptions associated with the approach as differing methods of analysis are based on differing assumptions about the data and how it will respond. Prevalence rates found in studies only provide estimates of the true prevalence of a problem in the larger population. Since some subgroups are very small, 95% confidence intervals are usually given.

9. Are all important confounding factors/ subgroups/differences identified and accounted for?

Incidence and prevalence studies often draw or report findings regarding the differences between groups. It is important that authors of these studies identify all important confounding factors, subgroups and differences and account for these.

10. Were subpopulations identified using objective criteria?

Objective criteria should also be used where possible to identify subgroups (refer to question 6).

If a study scores less than 5/10 (50%) it should be excluded, unless there is ambiguity with relation to the aforementioned criteria, in which case more information should be sought and then the criteria rereviewed.

For peer review only

	Study details								Study Methods											
													Subject characteristics							
Study	Author	Year of Publication	Study title	Journal of publication	Aim	Setting	Ward / Department / Unit / Hospital / Clinical population type	Study design	Recruitment duration	Age of participants (mean +/-SD)	Age of participants (range)	Sex (proportion male / female participants)	Country / location	Continent	5 year average GDP per capita PPP (current international $) (years proceeding the study) (International	5 year average healthcare expenditure per capita PPP (current international $) (years proceeding the study) (World	Sample size (n)	Diagnosis / Prevalent morbidity (if applicable)	Any other relevant characteristic	Criteria utilised for the operational definition of frailty
Study 2																				
Study 3																				
Study 4																				
Study 5																				
Study 6																				
Study 7																				
Study 8																				
Study 9																				
Study 10																				
Study 11																				
Study 12																				
Study 13																				
Study 14																				
Study 15																				
Study 16																				
Study 17																				
Study 18																				
Study 19																				
Study 20																				
Study 21																				
Study 22																				
Study 23																				

For peer review only

Results

Number of frail participants	Number of "pre-frail" participants	Number of robust / non-frail	Prevalence of frailty	Prevalence of "pre-frailty"	Prevalence of robust/non frail	Number of Male participants	Number of frail Male participants	Number of pre-frail Male participants	Number of non-frail / robust Male participants	Prevalence of frailty in Male participants	Prevalence of pre-frailty in Male participants	Prevalence of robust / non-frailty Male participants	Number of Female participants	Number of frail Female partcipants	Number of pre-frail Female participants	Number of non-frail / robust Female participants	Prevalence of frailty in Female participants	Prevalence of pre-frailty in Female participants	Prevalence of robust / non-frialty Female participants	Relevant authors comments	Relevant reviewers comments	Planned stratified

For peer review only

For peer review only - http://bmjopen.bmj.com/site/about/guidelines.xhtml

					5 year average GDP per capita PPP (current international $) (years proceeding the study*) (International Monetary Fund data)																		
Author	Year of Publication	Country / location	Recruitment start date	Recruitment end date	Year 5	Year 4	Year 3	Year 2	Year 1	Additional Year 1	Additional year 2	Additional year 3	Additional year 4	Additional year 5	Additional year 6	Additional year 7	Additional year 8	Additional year 9	Additional year 10	Additional year 11	Additional year 12	Years	5 year average GDP per capita PPP (current international $) (years proceeding the study*) (Internation Monetary Fund data)

*5 years prior to commencement of data collection for the study. Each calender year of the study will also be included provided recruitment continues through to > 6 months in the proceeding year.

For peer review only

					5 year average healthcare expenditure per capita PPP (current international $) (years proceeding the study) (World Health Organisation data)																			
Author	Year of Publication	Country / location	Recruitment start date	Recruitment end date	Year 5	Year 4	Year 3	Year 2	Year 1	Additional Year 1	Additional year 2	Additional year 3	Additional year 4	Additional year 5	Additional year 6	Additional year 7	Additional year 8	Additional year 9	Additional year 10	Additional year 11	Additional year 12	Years	5 year average health care expenditure per capita PPP (current international $) (years proceeding the study*) (World Health Organisation data)	

*5 years prior to commencement of data collection for the study. Each calender year of the study will also be included provided recruitment continues through to > 6 months in the proceeding year.

PRISMA-P (Preferred Reporting Items for Systematic review and Meta-Analysis Protocols) 2015 checklist: recommended items to address in a systematic review protocol*

Section and topic	Item No	Checklist item	Page no.
**ADMINISTRATIVE INFORMATION**			
Title:			
Identification	1a	Identify the report as a protocol of a systematic review	1
Update	1b	If the protocol is for an update of a previous systematic review, identify as such	
Registration	2	If registered, provide the name of the registry (such as PROSPERO) and registration number	2
Authors:			
Contact	3a	Provide name, institutional affiliation, e-mail address of all protocol authors; provide physical mailing address of corresponding author	1
Contributions	3b	Describe contributions of protocol authors and identify the guarantor of the review	1,11
Amendments	4	If the protocol represents an amendment of a previously completed or published protocol, identify as such and list changes; otherwise, state plan for documenting important protocol amendments	
Support:			
Sources	5a	Indicate sources of financial or other support for the review	11
Sponsor	5b	Provide name for the review funder and/or sponsor	11
Role of sponsor or funder	5c	Describe roles of funder(s), sponsor(s), and/or institution(s), if any, in developing the protocol	
**INTRODUCTION**			
Rationale	6	Describe the rationale for the review in the context of what is already known	4
Objectives	7	Provide an explicit statement of the question(s) the review will address with reference to participants, interventions, comparators, and outcomes (PICO)	4,5
**METHODS**			
Eligibility criteria	8	Specify the study characteristics (such as PICO, study design, setting, time frame) and report characteristics (such as years considered, language, publication status) to be used as criteria for eligibility for the review	4,5
Information sources	9	Describe all intended information sources (such as electronic databases, contact with study authors, trial registers or other grey literature sources) with planned dates of coverage	5,6,7
Search strategy	10	Present draft of search strategy to be used for at least one electronic database, including planned limits, such that it could be repeated	Appendix 1
Study records:			

For peer review only

Data management	11a	Describe the mechanism(s) that will be used to manage records and data throughout the review	6,7,8
Selection process	11b	State the process that will be used for selecting studies (such as two independent reviewers) through each phase of the review (that is, screening, eligibility and inclusion in meta-analysis)	6,7,8,9,10
Data collection process	11c	Describe planned method of extracting data from reports (such as piloting forms, done independently, in duplicate), any processes for obtaining and confirming data from investigators	8,9
Data items	12	List and define all variables for which data will be sought (such as PICO items, funding sources), any pre-planned data assumptions and simplifications	8
Outcomes and prioritization	13	List and define all outcomes for which data will be sought, including prioritization of main and additional outcomes, with rationale	9,10
Risk of bias in individual studies	14	Describe anticipated methods for assessing risk of bias of individual studies, including whether this will be done at the outcome or study level, or both; state how this information will be used in data synthesis	7,8
Data synthesis	15a	Describe criteria under which study data will be quantitatively synthesised	7,8
	15b	If data are appropriate for quantitative synthesis, describe planned summary measures, methods of handling data and methods of combining data from studies, including any planned exploration of consistency (such as I^2, Kendall's τ)	9,10
	15c	Describe any proposed additional analyses (such as sensitivity or subgroup analyses, meta-regression)	9,10
	15d	If quantitative synthesis is not appropriate, describe the type of summary planned	8
Meta-bias(es)	16	Specify any planned assessment of meta-bias(es) (such as publication bias across studies, selective reporting within studies)	10
Confidence in cumulative evidence	17	Describe how the strength of the body of evidence will be assessed (such as GRADE)	

*** It is strongly recommended that this checklist be read in conjunction with the PRISMA-P Explanation and Elaboration (cite when available) for important clarification on the items. Amendments to a review protocol should be tracked and dated. The copyright for PRISMA-P (including checklist) is held by the PRISMA-P Group and is distributed under a Creative Commons Attribution Licence 4.0.**

From: Shamseer L, Moher D, Clarke M, Ghersi D, Liberati A, Petticrew M, Shekelle P, Stewart L, PRISMA-P Group. Preferred reporting items for systematic review and meta-analysis protocols (PRISMA-P) 2015: elaboration and explanation. BMJ. 2015 Jan 2;349(jan02 1):g7647.

For peer review only

BMJ Open

Frailty Levels in Geriatric Hospital paTients (FLIGHT) - The prevalence of frailty amongst geriatric populations within hospital ward settings: A systematic review protocol

Journal:	*BMJ Open*
Manuscript ID	bmjopen-2019-030147.R3
Article Type:	Protocol
Date Submitted by the Author:	10-Jul-2019
Complete List of Authors:	Doody, Paul; University of Birmingham, School of Sport, Exercise and Rehabilitation Sciences<br>Aunger, Justin; University of Birmingham, School of Sport, Exercise and Rehabilitation Sciences<br>Asamane, Evans; University of Birmingham, School of Sport, Exercise and Rehabilitation Sciences<br>Greig, Carolyn; School of Sport, Exercise and Rehabilitation Sciences, University of Birmingham, Edgbaston, Birmingham, B15 2TT, UK; MRC-Arthritis Research UK Centre for Musculoskeletal Ageing Research<br>Lord, Janet; University of Birmingham, NIHR Surgical Reconstruction & Microbiology Research Centre<br>Whittaker, Anna; University of Birmingham, School of Sport, Exercise and Rehabilitation Sciences; University of Stirling
**Primary Subject Heading**:	Geriatric medicine
Secondary Subject Heading:	Geriatric medicine, Health economics
Keywords:	Frail, Geriatric, inpatient, older adult, prevalence, systematic review

SCHOLARONE™
Manuscripts

Frailty Levels in Geriatric Hospital paTients (FLIGHT) - The prevalence of frailty amongst geriatric populations within hospital ward settings: A systematic review protocol

Paul Doody[1], Justin A. Aunger[1], Evans A. Asamane[1], Carolyn A. Greig[1,2,3], Janet M. Lord[2], Anna C. Whittaker[1,4]

[1] School of Sport, Exercise and Rehabilitation Sciences, University of Birmingham, United Kingdom

[2] Institute of Inflammation and Ageing MRC-ARUK Centre for Musculoskeletal Ageing Research, University of Birmingham, United Kingdom

[3] NIHR Birmingham Biomedical Research Centre, University Hospitals Birmingham NHS Foundation Trust and University of Birmingham, United Kingdom

[4] Faculty of Health Sciences and Sport, University of Stirling, United Kingdom

Corresponding author: Paul Doody (p.d.doody@bham.ac.uk, +44 (0) 121 414 4125), School of Sport Exercise and Rehabilitation Sciences (G35), University of Birmingham, United Kingdom

For peer review only

For peer review only - http://bmjopen.bmj.com/site/about/guidelines.xhtml

Abstract

Introduction: Frailty is a common and clinically significant condition in geriatric populations, associated with adverse health outcomes such as hospitalisation, disability, and mortality. Although there are systematic reviews/meta-analyses assessing the prevalence of frailty in community-dwelling older adults, nursing home residents, and cancer and general surgery patients, there are none assessing the overall prevalence of frailty in geriatric hospital inpatients.

Methods and analysis: This review will systematically search and analyse the prevalence of frailty within geriatric hospital inpatients within the literature. A search will be employed on the platforms of Ovid, Web of Science, and databases of CINAHL Plus, SCOPUS, and the Cochrane Library. Any observational or experimental study design which utilises a validated operational definition of frailty, reports the prevalence of frailty, has a minimum age ≥ 65 years, attempts to assess the whole ward/clinical population, and occurs in hospital inpatients, will be included. Title and abstract and full-text screenings will be conducted by three reviewers. Methodological quality of eligible studies will be assessed utilising the Joanna Briggs Institute critical appraisal tool. Data extraction will be performed by two reviewers. If sufficient data are available, a meta-analysis synthesising pooled estimates of the prevalence of frailty and pre-frailty, as well as the prevalence of frailty stratified by age, sex, operational frailty definition, prevalent morbidities, ward type, and location, among older hospitalised in-patients will be conducted. Clinical heterogeneity will be assessed by two reviewers. Statistical heterogeneity will be assessed through a Cochran Q test, and an I^2 test performed to assess its magnitude.

Ethics and dissemination: Ethical approval was not required as primary data will not be collected. Findings will be disseminated through publication in peer reviewed open access scientific journals, public engagement events, conference presentations, and social media**.**

Trial Registration number: This study has been registered on PROSPERO (registration number 79202).

Strengths and limitations of this study:

For peer review only - http://bmjopen.bmj.com/site/about/guidelines.xhtml

- First review to systematically or exclusively assess the overall prevalence of frailty in geriatric hospital inpatients
- Will seek to provide stratified analysis of the prevalence of frailty based on age, sex, operational frailty definition, prevalent morbidities, ward type and location
- Three independent reviewers during screening phase; ensuring high internal reliability and consistency of included studies
- Will include only studies for which the full text is available in English, therefore will likely be relatively over-representative of Western nations (Europe, Australasia, and the Americas); although this is true of scientific publications in general.
- **Keywords**: department; frail; geriatric; hospital; inpatient; meta-analysis; older adult; prevalence; systematic review; ward.

For peer review only

For peer review only - http://bmjopen.bmj.com/site/about/guidelines.xhtml

Introduction

Frailty is a common and clinically significant condition within geriatric populations (1), predominantly due to its association with adverse health outcomes such as hospitalization, disability and mortality (1-6). Although there are systematic reviews and meta-analysis assessing the prevalence of frailty amongst community-dwelling older adults (7-10), nursing home residents (11), and cancer (12) and general surgery patients (13), presently there are no systematic reviews or meta-analysis which assess the overall prevalence of frailty among geriatric hospital inpatients. This constitutes an important gap in the literature which needs to be addressed and has important consequences. Such consequences include the tailoring of services within this setting to the needs of service users, for example, the potential implementation of exercise rehabilitation treatments within this setting for this cohort; with physical activity and exercise being proposed as potentially offering the best form of treatment for frail older adults (14), and shown to be capable of reducing, and even reversing frailty within older adults (15,16). Through providing a highly detailed analysis of the prevalence of frailty amongst older population within this setting, this review has the potential to aid in the facilitation of improvements in the planning and orientation of organisational structures and resources, to meet the needs of this population, and enhance the care of frail older adults in inpatient hospital settings.

Methods and Design

Review Aim:

The aim of this review is to systematically search and analyse the prevalence of frailty amongst geriatric populations (aged ≥ 65 years) within inpatient hospital settings within the literature. If a meta-analysis proves possible, the aim of this study is also to synthesise pooled estimates of the prevalence of frailty and pre-frailty, as well as the prevalence of frailty stratified by age, sex, operational frailty definition, prevalent morbidities, ward type and location (country and continent), among hospital in-patients.

For peer review only - http://bmjopen.bmj.com/site/about/guidelines.xhtml

Review Objectives:

1) To identify and compare studies reporting the prevalence of frailty within hospital ward settings.
2) To combine the extracted data to calculate the pooled overall prevalence of frailty in hospitalised geriatric in-patients.
3) To perform stratified analysis of the prevalence of frailty based on age, sex, operational frailty definition, prevalent morbidity and ward type in order to assess the relationship between frailty and these factors.

Eligibility criteria:

Inclusion criteria: all studies must have a minimum age of ≥ 65 years, use a clearly defined and validated operational definition for the classification of frailty (i.e. has been specifically validated for the assessment of frailty, either through comparison with existing validated tools, or its predictive value regarding negative health outcomes aligned with frailty), either assess (or attempt to assess) the whole ward, department, unit, hospital or specific clinical population, or employ some form of randomised selection of participants, occur within a hospital setting, in, or including, hospital in-patients (operationally defined as any patient admitted to hospital who remains overnight, or were initially expected to remain overnight), report the prevalence of frailty or provide sufficient data to allow the calculation of the prevalence of frailty. If a study examines a mixed cohort, only data relating to hospital in-patients will be included in the review.

Exclusion criteria: all studies not written in English, studies where the sample are not hospital inpatients (i.e. outpatients, day patients or community-dwelling individuals).

Information sources:

Searches will be conducted on the platforms of Ovid (incorporating the databases of Journals @Ovid full text, EMBASE, CAB abstracts, Ovid MEDLINE ® In process and other non-indexed citations, Ovid MEDLINE ®, and PyschINFO) and Web of Science (incorporating the databases of Science Citation Index Expanded (SCI-Expanded), Conference Proceedings Citation Index – Science (CRI-S), and Emerging

For peer review only - http://bmjopen.bmj.com/site/about/guidelines.xhtml

Sources Citation Index (ESCI)), and the databases of CINAHL Plus, SCOPUS, and the Cochrane Library databases (the Cochrane Database of Systematic Reviews (CDSR), the Cochrane Central Register of Controlled Trials (CENTRAL), the Cochrane Methodology Register (CMR), the Database of Abstracts of Reviews of Effect (DARE), Health Technology Assessment database (HTA) and the NHS Economic Evaluation Database (EED)).

Types of studies:

Any form of observational or experimental study design which assesses the prevalence of frailty and meets the above eligibility criteria. For longitudinal observational studies, and experimental studies, frailty scores and additional data will be extracted from baseline data, provided baseline data meets the above eligibility criteria.

Search Strategy:

The search strategy will be conducted on the two platforms of Ovid and Web of Science, as well as the databases of SCOPUS, CINAHL Plus, and the Cochrane Library databases (Appendix 1). These searches will encompass all available literature published prior to 21/11/2018.

Screening:

Prior to the commencement of title and abstract screening by the three independent reviewers, duplicates will be removed utilising EndNote X8.2. The reduced list of studies will be manually screened for the removal of any remaining duplicates. All reviewers will be provided with an instructional screening form (Appendix 2), and a .ris file containing all studies captured within database searches. The screening form will list the eligibility criteria and instructions on setting up the .ris file for screening within a reference manager.

For peer review only

The title and abstract of all studies will then be independently screened by the three reviewers, with each reviewer placing potentially eligible studies into a separate folder. Upon completion, potentially eligible studies from all three reviewers will be placed into a "master folder" and the results collated. Duplicates will be removed, leaving the final combined list of studies for the full text screening phase. All reviewers will then independently screen the full text of remaining studies utilising the screening form and maintain separate files for included and excluded studies (including reasons), as well as for studies for which the reviewer feels the need to contact the authors for clarification or additional information.

Upon completion, a full text screening master file (Appendix 3) will be formulated by the lead reviewer displaying each reviewer's full text screening decision for each study. All three reviewers will then meet to discuss the decisions of each study and endeavour to come to an agreement on studies for which there is not initial unanimous consensus. During this process a full list of included and excluded studies (with reasons), and studies for which reviewers agree to contact authors for additional information or clarification will be formed by the lead reviewer. The lead reviewer will then contact study authors and, upon receipt of clarification or additional information, will meet with reviewers to discuss the inclusion/exclusion of the study.

Manual screening will also be employed by reviewers and include the reference lists of all included studies, as well as excluded but potentially relevant studies or systematic reviews captured within the screening. As part of the grey literature search of this review, in process publications will also be searched and conference abstracts will be followed up with authors to ascertain if a full text relating to the data is available. Studies of the same cohort will be included only once, using the study which provides the most information about the cohort relevant to this review.

Assessment of methodological quality:

The quality of eligible studies from full text screening will be assessed by two reviewers independently using the Joanna Briggs Institute (JBI) critical appraisal tool for studies reporting prevalence data (17)

For peer review only - http://bmjopen.bmj.com/site/about/guidelines.xhtml

(Appendix 4). In the event of any discrepancies between the two reviewers, a consensus will be attempted to be reached by discussion. In the event a full consensus cannot be reached between the two reviewers after an exhaustive discussion, the opinion of a third reviewer will be obtained, and the proceeding majority consensus will be taken.

Data extraction:

Data extraction will be performed by two reviewers independently. In the event of any discrepancies between the two reviewers, a consensus will be attempted to be reached by discussion. In the event that a full consensus cannot be reached between the two reviewers after an exhaustive discussion, the opinion of a third reviewer will be obtained, and the proceeding majority consensus will be taken.

The following data, where available, will be extracted from all eligible studies (see Appendix 5 for template). If any data are not immediately available, the authors of the studies in question will be contacted in an attempt to retrieve all applicable data:

Study details: authors, year of publication, study title, journal of publication, aim. Study methods: setting, ward/department/unit/hospital type/clinical population, study design, recruitment duration, subject characteristics (age of participants (mean and standard deviation, range)), sex (proportion of male / female participants), country / continent, sample size, diagnosis / prevalent morbidity (if applicable), any other relevant characteristics), criteria utilised for the operational definition of frailty. Results: Number of frail participants, number of “pre-frail” participants, number of robust / non-frail participants, prevalence of frailty, prevalence of pre-frailty, prevalence of robustness / non-frailty, number of male participants, number of frail male participants, number of pre-frail male participants, number of non-frail / robust male participants, prevalence of frailty in male participants, prevalence of pre-frailty in male participants, prevalence of non-frailty / robustness in male participants, number of female participants, number of frail female participants, number of pre-frail female participants, number of non-frail / robust female participants, prevalence of frailty in female participants, prevalence of pre-frailty in female participants, prevalence of non-frailty / robustness in female participants, and finally authors comments and reviewers’ comments.

For peer review only - http://bmjopen.bmj.com/site/about/guidelines.xhtml

External to the studies, data will also be extracted with regard to the 5 year average Gross Domestic Product (GDP) per capita Purchasing Power Parity (PPP) (current international $) of the country in which each study takes place, incorporating the five years directly preceding the commencement of recruitment to the study (18). External data will also be extracted with regard to the 5 year average health care expenditure per capita PPP (current international $) of the country in which each study takes place, incorporating the five years directly preceding the commencement of recruitment to the study (19). Each calendar year of the study will also be included provided recruitment continues through to > 6 months in the preceding year.

Data synthesis:

Quantitative synthesis (Meta-Analysis): If a sufficient quantity of identified studies are comparable, a meta-analysis, pooling the aggregated data from each study, will be performed. Clinical heterogeneity will be assessed by two reviewers based on their judgement of the available data and any disagreements will be discussed thoroughly with the aim of reaching a unanimous consensus. If a unanimous consensus cannot be reached, the opinion of a third reviewer will be sought, and the proceeding majority consensus will be taken. Statistical heterogeneity will be assessed through the utilisation of a Cochran Q test and considered present at $p < .05$. An I^2 test will be performed in order to assess the magnitude of this heterogeneity, with I^2 values of 25%, 50% and 75% being considered low, moderate and high respectively. If the Cochrane Q statistic test detected statistically significant heterogeneity, combined with the researcher's assessment, a randomised-effects model will be utilised. Given the nature of this review and in particular its overall aim, combined with the eligible studies identified in preliminary searches, it is likely the initial quantitative synthesis will utilise a random-effects model.

Stratified analysis will also be conducted according to age (65 – 74 years, 75 – 84 years and 85+ years), sex, operational frailty definition, ward type, prevalent morbidity and location (country and continent) where possible. These variables have been specifically chosen for stratified analysis predominantly due to an enhanced knowledge of these areas being of practical utility to researchers and clinicians; stemming from empirical evidence persistently showing alterations in these factors to impact upon the prevalence of frailty

For peer review only - http://bmjopen.bmj.com/site/about/guidelines.xhtml

(2,4,20-22). As such stratified analysis pertaining to these variables will facilitate this review to provide a more in-depth and thorough insight into the prevalence of frailty amongst geriatric hospital inpatients.

Clinical heterogeneity for stratified analysis will be assessed by two reviewers based on their judgement of the available data. Any disagreements will be discussed thoroughly with the aim of reaching a unanimous consensus. If a unanimous consensus cannot be reached, the opinion of a third reviewer will be sought. Statistical heterogeneity for sub-analysis will similarly be assessed through the utilisation of a Cochran Q test and considered present at $p < .05$. An I^2 test will be performed in order to assess the magnitude of this heterogeneity, with I^2 values of 25%, 50% and 75% being considered low, moderate and high respectively.

Similarly, it is likely a random-effects model will be utilised to synthesise pooled estimates of the prevalence of frailty stratified by these criteria (although there is more of a likelihood that a fixed effects model could potentially be utilised within these analyses, in comparison to the initial analysis, given the nature of stratified analysis).

Correlation analysis will also be employed to examine the relationship between the prevalence of frailty of geriatric inpatients and economic prosperity (GDP per capita PPP) (current international $), and health care expenditure (per capita PPP) (current international $). Additionally, multi-linear regression analysis will examine the predictive value between economic prosperity and health care expenditure, and the prevalence of frailty of geriatric inpatients. Preliminary research into these areas have shown frailty in the community to be correlated with economic indicators (GDP per capita PPP) (23), however, note that more research is needed in this regard to better understand this relationship; which this review will facilitate through examination of the relationship of GDP per capita PPP and health care expenditure, and the prevalence of frailty amongst geriatric hospital inpatients.

Qualitative synthesis: if a meta-analysis is not possible based on the nature of the studies and the data available, a more thorough systematic narrative analysis will be conducted, with findings presented in both textual and tabular formats.

For peer review only - http://bmjopen.bmj.com/site/about/guidelines.xhtml

Patient and Public involvement

All authors are strong proponents of patient and public involvement and engagement with research and believe the finding of this review will be important to aid the facilitation of improvements in the planning and orientation of organisation structures and resources within this setting to meet the needs of service users; specifically relating to the enhanced care of older adults in inpatient hospital settings. However, given the nature of this study (systematic review), it was not possible to involve the public. However, the findings will be disseminated to our patient and public involvement groups.

Ethics and Dissemination

Formal ethical approval was not required for this review as primary data will not be collected. The findings of this study will be disseminated through publication in the form of scientific papers in peer reviewed open access scientific journals, public engagement events within the United Kingdom and Europe, online via social media (Twitter, Instagram) and the PANINI project website (24,25), and presentation at conferences within the UK and internationally. This review is scheduled for completion during the second half of 2019.

Funding

This review has been funded by the European Commission's Horizon 2020 research and innovation programme under the Marie Sklodowska-Curie grant agreement (675003); of which PD, EA and JA are Marie Sklodowska-Curie Doctoral Research Fellows, AW, JL and CG doctoral supervisors, and AW the grants Principal Investigator.

Author contributions

For peer review only - http://bmjopen.bmj.com/site/about/guidelines.xhtml

PD is guarantor and lead reviewer. PD designed the systematic review protocol, conducted the literature searches and prepared this manuscript for publication, with supervision, input and feedback from AW, CG and JL. EA and JA are independent reviewers for title and abstract and full text screenings. JA will also act as independent data extractor for included studies. All authors have read and approved the final manuscript.

Conflicts of interest:

The authors of this review report no known conflicts of interest.

Acknowledgements:

The authors of this review would like to thank and acknowledge Ms. Lynne Harris (Subject advisor of the Main Library at the University of Birmingham, United Kingdom) for her assistance during the formulation of the search strategy utilised within this systematic review.

Word count (excluding title page, abstract, author contributions, acknowledgements and references): 2507

For peer review only

References:

(1) Rodriguez-Mañas L, Fried LP. Frailty in the clinical scenario. The Lancet 2015 /02/14;385(9968):e9.

(2) Fried LP, Tangen CM, Walston J, Newman AB, Hirsch C, Gottdiener J, et al. Frailty in older adults evidence for a phenotype. The Journals of Gerontology Series A: Biological Sciences and Medical Sciences 2001;56(3):M157.

(3) Gill TM, Gahbauer EA, Allore HG, Han L. Transitions between frailty states among community-living older persons. Arch Intern Med 2006;166(4):418-423.

(4) Sternberg SA, Schwartz AW, Karunananthan S, Bergman H, Mark Clarfield A. The identification of frailty: a systematic literature review. J Am Geriatr Soc 2011;59(11):2129-2138.

(5) Clegg A, Young J, Iliffe S, Rikkert MO, Rockwood K. Frailty in elderly people. The Lancet 2013 March 8,;381(9868):752-762.

(6) Sourial N, Bergman H, Karunananthan S, Wolfson C, Payette H, Gutierrez-Robledo LM, et al. Implementing Frailty Into Clinical Practice: A Cautionary Tale. J Gerontol A Biol Sci Med Sci 2013 -05-02:glt053.

(7) Collard RM, Boter H, Schoevers RA, Oude Voshaar RC. Prevalence of frailty in community-dwelling older persons: a systematic review. J Am Geriatr Soc 2012 Aug;60(8):1487-1492.

(8) Siriwardhana DD, Hardoon S, Rait G, Weerasinghe MC, Walters KR. Prevalence of frailty and prefrailty among community-dwelling older adults in low-income and middle-income countries: a systematic review and meta-analysis. BMJ open 2018;8(3):e018195.

(9) Kojima G, Iliffe S, Taniguchi Y, Shimada H, Rakugi H, Walters K. Prevalence of frailty in Japan: A systematic review and meta-analysis. Journal of epidemiology 2017;27(8):347-353.

(10) Verlaan S, Ligthart-Melis GC, Wijers SL, Cederholm T, Maier AB, de van der Schueren, Marian AE. High prevalence of physical frailty among community-dwelling malnourished older adults–a systematic review and meta-analysis. Journal of the American Medical Directors Association 2017;18(5):374-382.

(11) Kojima G. Prevalence of frailty in nursing homes: A systematic review and meta-analysis. Journal of the American Medical Directors Association 2015;16(11):940-945.

(12) Handforth C, Clegg A, Young C, Simpkins S, Seymour MT, Selby PJ, et al. The prevalence and outcomes of frailty in older cancer patients: a systematic review. Annals of Oncology 2015 June;26(6):1091-1101.

(13) Hewitt J, Long S, Carter B, Bach S, McCarthy K, Clegg A. The prevalence of frailty and its association with clinical outcomes in general surgery: a systematic review and meta-analysis. Age Ageing 2018;47(6):793-800.

(14) Theou O, Stathokostas L, Roland KP, Jakobi JM, Patterson C, Vandervoort AA, et al. The effectiveness of exercise interventions for the management of frailty: a systematic review. Journal of aging research 2011;2011.

(15) Fiatarone MA, O'Neill EF, Ryan ND, Clements KM, Solares GR, Nelson ME, et al. Exercise training and nutritional supplementation for physical frailty in very elderly people. N Engl J Med 1994;330(25):1769-1775.

(16) Tarazona-Santabalbina FJ, Gmez-Cabrera MC, Prez-Ros P, Martnez-Arnau FM, Cabo H, Tsaparas K, et al. A multicomponent exercise intervention that reverses frailty and improves cognition, emotion, and social networking in the community-dwelling frail elderly: a randomized clinical trial. Journal of the American Medical Directors Association 2016;17(5):426-433.

For peer review only - http://bmjopen.bmj.com/site/about/guidelines.xhtml

(17) Munn Z, Moola S, Riitano D, Lisy K. The development of a critical appraisal tool for use in systematic reviews addressing questions of prevalence. International journal of health policy and management 2014;3(3):123.

(18) International Monetary Fund (. World Economic Outlook (April 2019). 2019; Available at: https://www.imf.org/external/datamapper/PPPPC@WEO/ADVEC/WEOWORLD. Accessed 02/05/, 2019.

(19) World Health Organisation (. Global Health Expenditure Database (GHED). 2019; Available at: http://apps.who.int/nha/database/Home/Index/en. Accessed 02/05/, 2019.

(20) Purser JL, Kuchibhatla MN, Fillenbaum GG, Harding T, Peterson ED, Alexander KP. Identifying frailty in hospitalized older adults with significant coronary artery disease. J Am Geriatr Soc 2006;54(11):1674-1681.

(21) Santos-Eggimann B, Cuénoud P, Spagnoli J, Junod J. Prevalence of frailty in middle-aged and older community-dwelling Europeans living in 10 countries. The Journals of Gerontology: Series A 2009;64(6):675-681.

(22) Andela RM, Dijkstra A, Slaets JPJ, Sanderman R. Prevalence of frailty on clinical wards: Description and implications; 20158543. Int J Nurs Pract 2010;16(1):14-19.

(23) Theou O, Brothers TD, Rockwood MR, Haardt D, Mitnitski A, Rockwood K. Exploring the relationship between national economic indicators and relative fitness and frailty in middle-aged and older Europeans. Age Ageing 2013;42(5):614-619.

(24) Whittaker AC, Delledonne M, Finni T, Garagnani P, Greig C, Kallen V, et al. Physical Activity and Nutrition INfluences In ageing (PANINI): consortium mission statement. Aging clinical and experimental research 2018:1-8.

(25) University of Birmingham. Physical Activity and Nutritional INfluences In ageing (PANINI) Available at: http://www.birmingham.ac.uk/generic/panini/index.aspx. Accessed 6th February, 2019.

For peer review only

Search Strategy:

Ovid Search Strategy

1. Frail$.ti.ab.
2. Prevalence.ti,ab.
3. Percent$.ti,ab.
4. "were frail".ti,ab.
5. "considered frail".ti,ab.
6. Hospital$.ti,ab.
7. Ward.ti,ab.
8. Department.ti,ab.
9. Surg*.ti,ab.
10. Unit.ti,ab.
11. Geriatr*.tx.
12. "older adult*".tx.
13. Elder$.tx.
14. Retire*.tx.
15. Old$.tx.
16. Patient$.tx.
17. "community-dwelling".ti,ab.
18. 2 OR 3 OR 4 OR 5
19. 6 OR 7 OR 8 OR 9 OR 10
20. 11 OR 12 OR 13 OR 14 OR 15 OR 16
21. 1 AND 18 AND 19 AND 20
22. 21 NOT 17

Scopus Search Strategy

((((TITLE-ABS-KEY(frail*)) AND (TITLE-ABS-KEY(Prevalence)) OR (TITLE-ABS-KEY(Percent*)) OR (TITLE-ABS-KEY ("were frail")) OR (TITLE-ABS-KEY ("considered frail"))) AND (((TITLE-ABS-KEY(Hospital*)) OR (TITLE-ABS-KEY(Ward)) OR (TITLE-ABS-KEY(Department)) OR (TITLE-ABS-KEY(surg*)) OR (TITLE-ABS-KEY(unit))))) AND ((ALL(Geriatr*)) OR (ALL("older adult*")) OR (ALL(Elder*)) OR (ALL(retire*)) OR (ALL(old)) OR (ALL(older)) OR (ALL(Patient*)))) AND NOT (TITLE-ABS-KEY("community-dwelling"))

Web of Science Search Strategy

1. TS = Frail*
2. TS = Prevalence
3. TS = Percent*
4. TS = "were frail"
5. TS = "considered frail"
6. TS = Hospital*
7. TS = Ward
8. TS = Department
9. TS = Surg*
10. TS = Unit
11. TS = Geriatr*
12. TS = "older adult"
13. TS = Elder*
14. TS = Retir*
15. TS = Old*
16. TS = Patient*
17. TS = "community-dwelling"
18. #2 OR #3 OR #4 OR #5

For peer review only

19. #6 OR #7 OR #8 OR #9 OR #10
20. #11 OR #12 OR #13 OR #14 OR #15 OR #16
21. #1 AND #18 AND #19 AND #20
22. #21 NOT #17

CINAHL PLUS Search Strategy

1. AB frail*
2. AB prevalence OR AN Percent* OR AB "were frail" OR AB "considered frail"
3. AB Hospital* OR AB Ward OR AB Department OR AB Surg* OR AB Unit
4. AB Geriatr* OR AB "older adult" OR AB Elder* OR AB Retir* OR AB OLD* OR AB Patient*
5. S1 AND S2 AND S3 AND S4

Cochrane Library Search Strategy

1. frail*:ti,ab,kw (Word variations have been searched)
2. prevalence:ti,ab,kw or percent*:ti,ab,kw or "were frail":ti,ab,kw or "considered frail":ti.ab.kw (Word variations have been searched)
3. hospital*:ti,ab,kw or ward:ti,ab,kw or department:ti,ab,kw or surg*:ti,ab,kw or unit:ti,ab,kw (Word variations have been searched)
4. Geriatr*:ti,ab,kw or "older adult":ti,ab,kw or Elder*:ti,ab,kw or Retir*:ti,ab,kw or Old*:ti,ab,kw (Word variations have been searched)
5. Patient*:ti,ab,kw (Word variations have been searched)
6. #4 OR #5
7. #1 AND #2 AND #3 AND #6

For peer review only

Frailty Levels In Geriatric Hospital in-paTients (FLIGHT)
Systematic Review Search Strategy Screening form
PANINI (Physical Activity and Nutritional INfluences in Ageing) project
University of Birmingham
2016 - 2019

UNIVERSITY OF BIRMINGHAM

"The prevalence of frailty amongst geriatric populations within hospital ward settings: A systematic review"

Inclusion criteria - All studies must:

- have a minimum age of ≥ 65 years
- use a clearly defined and validated operational definition for the classification of frailty
- either assess (or attempt to assess) the whole ward, department, unit, hospital, or clinical population, or employ some form of randomised selection of participants
- occur within a hospital setting, in, or including hospital in-patients*
- report the prevalence of frailty or provide sufficient data to allow the calculation of the prevalence of frailty.

* If a study examines a mixed cohort, only data relating to hospital in-patients will be included.

Exclusion criteria:

- studies not written in English
- studies where the sample are non-hospital in-patients (i.e. outpatients, day patients or community-dwelling individuals)

Systematic Review - Screening procedure

1). Import attached RIS file into your reference manager software (preferably EndNote X8.2)

2). Once imported, scan all title and abstracts for eligibility against the inclusion / exclusion criteria above.

3). Move all studies identified as potentially eligible based on title and abstract into a separate group (EndNote), Or folder (RefWorks, Mendeley).

4). Screen full text of identified studies to determine eligibility.

5). Move all eligible studies into separate group / folder.

6). Make note of excluded studies and reasons for their exclusion based on eligibility criteria, in the attached excel file.

5). Compare identified studies.

6). If all reviewers identify the exact same studies, with no discrepancies, this is the end of the initial screening process for the systematic review.

7). If there are differences in the studies identified by different reviewers - discuss until resolution is determined. In the event a unanimous consensus cannot be met by the three reviewers, the majority consensus will be taken, and a note made of this.

For peer review only - http://bmjopen.bmj.com/site/about/guidelines.xhtml

Author	Title	Initial studies included from full text screening (prior to reviewer discussion)		
		Reviewer 1 (PD)	Reviewer 2 (JA)	Reviewer 3 (EA)

For peer review only

Conclusion of discussion	Decision	Response received from author	Outcome

For peer review only

Key

**Unanimous consensus inclusion**	
**Majority consensus inclusion**	
**Minority consensus inclusion / contact author***	*In event where either there is not majority consensus inclusion or exclusion i.e. one reviewer wishes to include and at least one other wishes to seek further information, or two reviewers wish to seek futher information
**Majority consensus exclusion****	** = Reasons for all excluded studies are given in exclusion form
**Unanimous consensus exclusion****	** = Reasons for all excluded studies are given in exclusion form
Unanimous consensus contact author	
? = Contact author***	** = Reasons for all instances of contacting the study author for clarification or futher information to assess eligibility are outlined in contact author form

✓ = Included

x = Excluded

For peer review only

JBI Critical Appraisal Checklist for Studies Reporting Prevalence Data

Reviewer: ______________________________ Date: ______________________________

Author: ______________________________ Year: __________ Record Number: __________

	Yes	No	Unclear	Not applicable
1. Was the sample representative of the target population?	☐	☐	☐	☐
2. Were study participants recruited in an appropriate way?	☐	☐	☐	☐
3. Was the sample size adequate?	☐	☐	☐	☐
4. Were the study subjects and the setting described in detail?	☐	☐	☐	☐
5. Was the data analysis conducted with sufficient coverage of the identified sample?	☐	☐	☐	☐
6. Were objective, standard criteria used for the measurement of the condition?	☐	☐	☐	☐
7. Was the condition measured reliably?	☐	☐	☐	☐
8. Was there appropriate statistical analysis?	☐	☐	☐	☐
9. Are all important cofounding factors/ subgroups/ differences identified and accounted for?	☐	☐	☐	☐
10. Were subpopulations identified using objective criteria?	☐	☐	☐	☐

Overall appraisal: Include ☐ Exclude ☐ Seek further info ☐

____

____

____

____

For peer review only

Critical appraisal tool guidelines

Answer: Yes, No, Unclear or Not/Applicable.

1. Was the sample representative of the target population?

This question relies upon knowledge of the broader characteristics of the population of interest. If the study is of women with breast cancer, knowledge of at least the characteristics, demographics and medical history is needed. The term "target population" should not be taken to infer every individual from everywhere or with similar disease or exposure characteristics. Instead, give consideration to specific population characteristics in the study, including age range, gender, morbidities, medications, and other potentially influential factors. For example, a sample may not be representative of the target population if a certain group has been used (such as those working for one organisation, or one profession) and the results then inferred to the target population (i.e. working adults).

2. Were study participants recruited in an appropriate way?

Recruitment is the calling or advertising strategy for gaining interest in the study, and is not the same as sampling. Studies may report random sampling from a population, and the methods section should report how sampling was performed. What source of data were study participants recruited from? Was the sampling frame appropriate? For example, census data is a good example of appropriate recruitment as a good census will identify everybody. Was everybody included who should have been included? Were any groups of persons excluded? Was the whole population of interest surveyed? If not, was random sampling from a defined subset of the population employed? Was stratified random sampling with eligibility criteria used to ensure the sample was representative of the population that the researchers were generalizing to?

3. Was the sample size adequate?

An adequate sample size is important to ensure good precision of the final estimate. Ideally we are looking for evidence that the authors conducted a sample size calculation to determine an adequate sample size. This will estimate how many subjects are needed to produce a reliable estimate of the measure(s) of interest. For conditions with a low prevalence, a larger sample size is needed. Also consider sample sizes for subgroup (or characteristics) analyses, and whether these are appropriate. Sometimes, the study will be large enough (as in large national surveys) whereby a sample size calculation is not required. In these cases, sample size can be considered adequate.

When there is no sample size calculation and it is not a large national survey, the reviewers may consider conducting their own sample size analysis using the following formula:[15, 16]

$N = Z2P(1-P)$

$d2$

Where:

- N = sample size
- Z = Z statistic for a level of confidence
- P = Expected prevalence or proportion (in proportion of one; if 20%, P = 0.2)
- d = precision (in proportion of one; if 5%, d=0.05)

4. Were the study subjects and setting described in detail?

Certain diseases or conditions vary in prevalence across different geographic regions and populations (e.g. women vs. men, sociodemographic variables between countries). Has the study sample been described in sufficient detail so that other researchers can determine if it is comparable to the population of interest to them?

5. Is the data analysis conducted with sufficient coverage of the identified sample?

A large number of dropouts, refusals or "not founds" amongst selected subjects may diminish a study's validity, as can low response rates for survey studies.

- Did the authors describe the reasons for non-response and compare persons in the study to those not in the study, particularly with regards to their socio-demographic characteristics?

For peer review only

- Could the not-responders have led to an underestimate of prevalence of the disease or condition under investigation?
- If reasons for non-response appear to be unrelated to the outcome measured and the characteristics of non-responders are comparable to those in the study, the researchers may be able to justify a more modest response rate.
- Did the means of assessment or measurement negatively affect the response rate (measurement should be easily accessible, conveniently timed for participants, acceptable in length and suitable in content).

6. Were objective, standard criteria used for measurement of the condition?

Here we are looking for measurement or classification bias. Many health problems are not easily diagnosed or defined, and some measures may not be capable of including or excluding appropriate levels or stages of the health problem. If the outcomes were assessed based on existing definitions or diagnostic criteria, then the answer to this question is likely to be yes. If the outcomes were assessed using observer reported, or self-reported scales, the risk of over-or under-reporting is increased, and objectivity is compromised. Importantly, determine if the measurement tools used were validated instruments as this has a significant impact on outcome assessment validity.

7. Was the condition measured reliably?

Considerable judgment is required to determine the presence of some health outcomes. Having established the objectivity of the outcome measurement instrument (see item 6 of this scale), it is important to establish how the measurement was conducted. Were those involved in collecting data trained or educated in the use of the instrument/s? If there was more than one data collector, were they similar in terms of level of education, clinical or research experience, or level of responsibility in the piece of research being appraised?
- Has the researcher justified the methods chosen?
- Has the researcher made the methods explicit? (For interview method, how were interviews conducted?)

8. Was there appropriate statistical analysis?

As with any consideration of statistical analysis, consideration should be given to whether there was a more appropriate alternate statistical method that could have been used. The methods section should be detailed enough for reviewers to identify the analytical technique used and how specific variables were measured. Additionally, it is also important to assess the appropriateness of the analytical strategy in terms of the assumptions associated with the approach as differing methods of analysis are based on differing assumptions about the data and how it will respond. Prevalence rates found in studies only provide estimates of the true prevalence of a problem in the larger population. Since some subgroups are very small, 95% confidence intervals are usually given.

9. Are all important confounding factors/ subgroups/differences identified and accounted for?

Incidence and prevalence studies often draw or report findings regarding the differences between groups. It is important that authors of these studies identify all important confounding factors, subgroups and differences and account for these.

10. Were subpopulations identified using objective criteria?

Objective criteria should also be used where possible to identify subgroups (refer to question 6).

If a study scores less than 5/10 (50%) it should be excluded, unless there is ambiguity with relation to the aforementioned criteria, in which case more information should be sought and then the criteria rereviewed.

For peer review only

			Study details									Study Methods			Subject characteristics					
Study	Author	Year of Publication	Study title	Journal of publication	Aim	Setting	Ward / Department / Unit / Hospital / Clinical population type	Study design	Recruitment duration	Age of participants (mean +/-SD)	Age of participants (range)	Sex (proportion male / female participants)	Country / location	Continent	5 year average GDP per capita PPP (current international $) (years proceeding the study) (International	5 year average healthcare expenditure per capita PPP (current international $) (years proceeding the study) (World	Sample size (n)	Diagnosis / Prevalent morbidity (if applicable)	Any other relevant characteristic	Criteria utilised for the operational definition of frailty
Study 2																				
Study 3																				
Study 4																				
Study 5																				
Study 6																				
Study 7																				
Study 8																				
Study 9																				
Study 10																				
Study 11																				
Study 12																				
Study 13																				
Study 14																				
Study 15																				
Study 16																				
Study 17																				
Study 18																				
Study 19																				
Study 20																				
Study 21																				
Study 22																				
Study 23																				

For peer review only

BMJ Open

Results																						Planned stratified
Number of frail participants	Number of "pre-frail" participants	Number of robust / non-frail	Prevalence of frailty	Prevalence of "pre-frailty"	Prevalence of robust/non frail	Number of Male participants	Number of frail Male participants	Number of pre-frail Male participants	Number of non-frail / robust Male participants	Prevalence of frailty in Male participants	Prevalence of pre-frailty in Male participants	Prevalence of robust / non-frailty Male participants	Number of Female participants	Number of frail Female parcipants	Number of pre-frail Female participants	Number of non-frail / robust Female participants	Prevalence of frailty in Female participants	Prevalence of pre-frailty in Female participants	Prevalence of robust / non-frialty Female participants	Relevant authors comments	Relevant reviewers comments	

For peer review only

For peer review only - http://bmjopen.bmj.com/site/about/guidelines.xhtml

					5 year average GDP per capita PPP (current international $) (years proceeding the study*) (International Monetary Fund data)																		
Author	Year of Publication	Country / location	Recruitment start date	Recruitment end date	Year 5	Year 4	Year 3	Year 2	Year 1	Additional Year 1	Additional year 2	Additional year 3	Additional year 4	Additional year 5	Additional year 6	Additional year 7	Additional year 8	Additional year 9	Additional year 10	Additional year 11	Additional year 12	Years	5 year average GDP per capita PPP (current international $) (years proceeding the study*) (Internation Monetary Fund data)

*5 years prior to commencement of data collection for the study. Each calender year of the study will also be included provided recruitment continues through to > 6 months in the proceeding year.

For peer review only

					5 year average healthcare expenditure per capita PPP (current international $) (years proceeding the study) (World Health Organisation data)																				
Author	Year of Publicatio n	Country / location	Recruitment start date	Recruitment end date	Year 5	Year 4	Year 3	Year 2	Year 1	Additional Year 1	Additional year 2	Additional year 3	Additional year 4	Additional year 5	Additional year 6	Additional year 7	Additional year 8	Additional year 9	Additional year 10	Additional year 11	Additional year 12	Years	5 year average health care expenditure per capita PPP (current international $) (years proceeding the study*) (World Health Organisation data)	

*5 years prior to commencement of data collection for the study. Each calender year of the study will also be included provided recruitment continues through to > 6 months in the proceeding year.

For peer review only

PRISMA-P (Preferred Reporting Items for Systematic review and Meta-Analysis Protocols) 2015 checklist: recommended items to address in a systematic review protocol*

Section and topic	Item No	Checklist item	Page no.
**ADMINISTRATIVE INFORMATION**			
Title:			
Identification	1a	Identify the report as a protocol of a systematic review	1
Update	1b	If the protocol is for an update of a previous systematic review, identify as such	
Registration	2	If registered, provide the name of the registry (such as PROSPERO) and registration number	2
Authors:			
Contact	3a	Provide name, institutional affiliation, e-mail address of all protocol authors; provide physical mailing address of corresponding author	1
Contributions	3b	Describe contributions of protocol authors and identify the guarantor of the review	1,11
Amendments	4	If the protocol represents an amendment of a previously completed or published protocol, identify as such and list changes; otherwise, state plan for documenting important protocol amendments	
Support:			
Sources	5a	Indicate sources of financial or other support for the review	11
Sponsor	5b	Provide name for the review funder and/or sponsor	11
Role of sponsor or funder	5c	Describe roles of funder(s), sponsor(s), and/or institution(s), if any, in developing the protocol	
**INTRODUCTION**			
Rationale	6	Describe the rationale for the review in the context of what is already known	4
Objectives	7	Provide an explicit statement of the question(s) the review will address with reference to participants, interventions, comparators, and outcomes (PICO)	4,5
**METHODS**			
Eligibility criteria	8	Specify the study characteristics (such as PICO, study design, setting, time frame) and report characteristics (such as years considered, language, publication status) to be used as criteria for eligibility for the review	4,5
Information sources	9	Describe all intended information sources (such as electronic databases, contact with study authors, trial registers or other grey literature sources) with planned dates of coverage	5,6,7
Search strategy	10	Present draft of search strategy to be used for at least one electronic database, including planned limits, such that it could be repeated	Appendix 1
Study records:			

For peer review only

Data management	11a	Describe the mechanism(s) that will be used to manage records and data throughout the review	6,7,8
Selection process	11b	State the process that will be used for selecting studies (such as two independent reviewers) through each phase of the review (that is, screening, eligibility and inclusion in meta-analysis)	6,7,8,9,10
Data collection process	11c	Describe planned method of extracting data from reports (such as piloting forms, done independently, in duplicate), any processes for obtaining and confirming data from investigators	8,9
Data items	12	List and define all variables for which data will be sought (such as PICO items, funding sources), any pre-planned data assumptions and simplifications	8
Outcomes and prioritization	13	List and define all outcomes for which data will be sought, including prioritization of main and additional outcomes, with rationale	9,10
Risk of bias in individual studies	14	Describe anticipated methods for assessing risk of bias of individual studies, including whether this will be done at the outcome or study level, or both; state how this information will be used in data synthesis	7,8
Data synthesis	15a	Describe criteria under which study data will be quantitatively synthesised	7,8
	15b	If data are appropriate for quantitative synthesis, describe planned summary measures, methods of handling data and methods of combining data from studies, including any planned exploration of consistency (such as I^2, Kendall's τ)	9,10
	15c	Describe any proposed additional analyses (such as sensitivity or subgroup analyses, meta-regression)	9,10
	15d	If quantitative synthesis is not appropriate, describe the type of summary planned	8
Meta-bias(es)	16	Specify any planned assessment of meta-bias(es) (such as publication bias across studies, selective reporting within studies)	10
Confidence in cumulative evidence	17	Describe how the strength of the body of evidence will be assessed (such as GRADE)	

*** It is strongly recommended that this checklist be read in conjunction with the PRISMA-P Explanation and Elaboration (cite when available) for important clarification on the items. Amendments to a review protocol should be tracked and dated. The copyright for PRISMA-P (including checklist) is held by the PRISMA-P Group and is distributed under a Creative Commons Attribution Licence 4.0.**

From: Shamseer L, Moher D, Clarke M, Ghersi D, Liberati A, Petticrew M, Shekelle P, Stewart L, PRISMA-P Group. Preferred reporting items for systematic review and meta-analysis protocols (PRISMA-P) 2015: elaboration and explanation. BMJ. 2015 Jan 2;349(jan02 1):g7647.

For peer review only
